# Utilizing VSWIR spectroscopy for macronutrient and micronutrient profiling in winter wheat

**DOI:** 10.3389/fpls.2024.1426077

**Published:** 2024-10-31

**Authors:** Anmol Kaur Gill, Srishti Gaur, Clay Sneller, Darren T. Drewry

**Affiliations:** ^1^ Department of Food, Agricultural, and Biological Engineering, Ohio State University, Columbus, OH, United States; ^2^ Department of Horticulture and Crop Science, Ohio State University, Wooster, OH, United States; ^3^ Department of Horticulture and Crop Science, Ohio State University, Columbus, OH, United States; ^4^ Translational Data Analytics Institute, Ohio State University, Columbus, OH, United States

**Keywords:** hyperspectral reflectance spectroscopy, VSWIR, winter wheat, macronutrients, micronutrients, partial least squares regression, PLSR, model selection

## Abstract

This study explores the use of leaf-level visible-to-shortwave infrared (VSWIR) reflectance observations and partial least squares regression (PLSR) to predict foliar concentrations of macronutrients (nitrogen, phosphorus, potassium, calcium, magnesium, and sulfur), micronutrients (boron, copper, iron, manganese, zinc, molybdenum, aluminum, and sodium), and moisture content in winter wheat. A total of 360 fresh wheat leaf samples were collected from a wheat breeding population over two growing seasons. These leaf samples were used to collect VSWIR reflectance observations across a spectral range spanning 350 to 2,500 nm. These samples were then processed for nutrient composition to allow for the examination of the ability of reflectance to accurately model diverse chemical components in wheat foliage. Models for each nutrient were developed using a rigorous cross-validation methodology in conjunction with three distinct component selection methods to explore the trade-offs between model complexity and performance in the final models. We examined absolute minimum predicted residual error sum of squares (PRESS), backward iteration over PRESS, and Van der Voet’s randomized *t*-test as component selection methods. In addition to contrasting component selection methods for each leaf trait, the importance of spectral regions through variable importance in projection scores was also examined. In general, the backward iteration method provided strong model performance while reducing model complexity relative to the other selection methods, yielding *R* ^2^ [relative percent difference (RPD), root mean squared error (RMSE)] values in the validation dataset of 0.84 (2.45, 6.91), 0.75 (1.97, 18.67), 0.78 (2.13, 16.49), 0.66 (1.71, 17.13), 0.68 (1.75, 14.51), 0.66 (1.72, 12.29), and 0.84 (2.46, 2.20) for nitrogen, calcium, magnesium, sulfur, iron, zinc, and moisture content on a wet basis, respectively. These model results demonstrate that VSWIR reflectance in combination with modern statistical modeling techniques provides a powerful high throughput method for the quantification of a wide range of foliar nutrient contents in wheat crops. This work has the potential to advance rapid, precise, and nondestructive field assessments of nutrient contents and deficiencies for precision agricultural management and to advance breeding program assessments.

## 1 Introduction

High-throughput phenotyping (HTP) offers tremendous potential to significantly improve breeding efficiency by expanding the traits used for selection to quantitative metrics of plant health and vigor (Araus and Cairns, 2014). Visible-to-shortwave infrared (VSWIR) spectroscopy is a method that measures surface reflectance across a spectral range spanning 350 to 2,500 nm. This technique encompasses the visible (400–700 nm), near-infrared (NIR) (700–1,000 nm), and shortwave infrared (1,000–2,500 nm) portions of the electromagnetic spectrum (Thompson et al., 2022). VSWIR sensing has been shown to be a powerful tool for vegetation monitoring (Im and Jensen, 2008; L. Singh et al., 2022), including for the quantification of foliar nutrient contents (Acosta et al., 2023; Lyu et al., 2023), making it a key technology in recent developments in many areas of HTP (Shakoor et al., 2017). Factors such as leaf surface properties, leaf internal structure, plant stress, and biochemical compounds impact reflectance, offering valuable insights into various aspects of plant functionality (Asner, 2008; Kokaly et al., 2009; Ustin and Jacquemoud, 2020). Nutritional stresses can cause an increase in the reflectance in the visible and infrared ranges, shifting the red edge to shorter wavelengths due to loss of chlorophyll (Zhao et al., 2005). The amount of change in spectral properties depends on the type and level of deficiency and also the interaction between chemical components (Ayala-Silva and Beyl, 2005).

A key challenge in both HTP and precision agriculture is the assessment of plant nutritional status. Macronutrients like nitrogen, phosphorus, potassium, calcium, magnesium, and sulfur exert distinct effects on plant growth (Hawkesford et al., 2023). Nitrogen is widely known to be a key factor in foliar chlorophyll content and photosynthetic capacity (Wang et al., 2021). Phosphorus contributes to seed formation, germination, energy storage, and cell growth (Khan et al., 2023). Potassium is crucial for stalk strength and various biochemical functions such as osmoregulation, enzyme activation, and the accumulation of proteins, carbohydrates, and fats (Wang et al., 2013). Calcium is involved in cell wall development, cell division, and increasing dry matter and leaf area (White and Broadley, 2003). Magnesium helps in photosynthesis and chlorophyll formation (Ahmed et al., 2023). Sulfur plays a fundamental role in constructing proteins and is a crucial component in the formation of chlorophyll (Fan et al., 2021; Weissert and Kehr, 2017). Micronutrients such as boron, copper, iron, manganese, zinc, molybdenum, aluminum, and sodium are typically required in smaller quantities but play a vital role in enhancing crop yield and quality (Broadley et al., 2012; Fageria et al., 2002). Insufficient levels of these nutrients can lead to stunted plant growth, organ damage, and even plant mortality (Gurudatta and Sachan, 2020; Pandey et al., 2020). Changes in plant nutrient availability can occur quickly and may be difficult to ameliorate, making it important to develop technologies capable of quantifying plant nutrient contents rapidly and remotely. Standard methods for plant tissue nutrient analysis include inductive coupled plasma–optical emission spectrometry for P, B, K, Ca, Mg, Cu, Zn, and Na; atomic absorption spectrometry for K, Ca, Mg, Cu, Zn, and Fe; Kjeldahl distillation for N; dry combustion for C, N, and S; and ultraviolet (UV)–visible spectrophotometry for Nitrate-N and P (Kalra, 1997; Menesatti et al., 2010). However, these traditional nutrient measurement methods are time-consuming, destructive, and expensive (Prananto et al., 2020). Utilizing leaf optical properties as an alternative provides a non-invasive, rapid, and cost-effective means to capture the spatial and temporal variations across an experimental field site or farm, providing a solution for site-specific precision fertilizer application (L. Singh et al., 2022; Zahir et al., 2024). This technology has the potential to dramatically improve precision agriculture and crop breeding assessments.

Partial least squares regression (PLSR) has been shown to be a powerful statistical modeling approach for utilizing the information contained in VSWIR spectroscopy signals while mitigating the problems around multi-collinearity in datasets with highly correlated variables (Chan et al., 2022). Examples of the successful application of PLSR to diagnose vegetation foliar states include its use in predicting leaf mass per area (Cherif et al., 2023; Doughty et al., 2011; Gara et al., 2019; Serbin et al., 2019), leaf area index (Cherif et al., 2023; Gara et al., 2019; Panigrahi and Das, 2021), and a range of chemical concentrations such as nitrogen (Doughty et al., 2011; Wang et al., 2015), carbon (Cherif et al., 2023; Gara et al., 2019; Serbin et al., 2014), lignin (Cherif et al., 2023; Singh et al., 2015), cellulose (Cherif et al., 2023; Serbin et al., 2014), hemicellulose (Asner et al., 2014; Kothari et al., 2023), soluble cell components (Serbin et al., 2014), phosphorus (Wang et al., 2015), potassium (Cherif et al., 2023), calcium (Asner et al., 2014; Kothari et al., 2023; Wang et al., 2020), magnesium (Asner et al., 2014; Kothari et al., 2023; Wang et al., 2020), and chlorophylls and carotenoids (Kothari et al., 2023; Serbin et al., 2014). Plant biotic and abiotic stress detection has also been estimated using PLSR (Adams et al., 2000; Asner et al., 2016; Žibrat et al., 2020). Leaf concentrations of nitrogen, phosphorus, potassium, calcium, and magnesium were modeled using hyperspectral data collected at pinot noir commercial vineyards in Martinborough, New Zealand (Lyu et al., 2023). PLSR has been shown to be versatile at predicting macro- and micronutrient contents in a variety of plant species including mangoes (Mahajan et al., 2021), citrus (Acosta et al., 2023), eucalyptus (Oliveira and Santana, 2020), and tef plant (Flynn et al., 2020); a combination of crops such as rice, corn, sesame, soybeans, tea, and grass (Zhai et al., 2012); and loblolly pine at local and regional scales (Stein et al., 2014).

In addition to PLSR, a linear regression method, non-linear regression techniques have also been successfully applied to estimate foliar nutrients. Non-linear machine learning models, such as Support Vector Regression (SVR), Random Forest Regression (RFR), Artificial Neural Networks (ANNs), Deep Neural Networks (DNNs), and Gaussian Process Regression (GPR), have gained prominence in modeling foliar nutrients in various fruits and row crops, including mango (Mahajan et al., 2021), cashew (Mahajan et al., 2024), orange (Osco et al., 2020b), apples (Azadnia et al., 2023), grapevines (Lyu et al., 2023), wheat (Jamali et al., 2023), barley (Grieco et al., 2022), soybeans (Furlanetto et al., 2023), and maize (Osco et al., 2020a). In several cases, these models have achieved superior predictive accuracy to PLSR, leading to more informed crop management decisions. For example, in a study on apple trees using visible and NIR spectroscopy, RFR model outperformed linear models, yielding relative percent difference (RPD) values of 8.77, 6.42, and 8.16 for N, P, and K, respectively (Azadnia et al., 2023). Similarly, in a study on nutrient assessment of mixed pastures in New Zealand, RFR provided the highest accuracy for N, P, K, Zn, Na, Cu, and Mg (*R* ^2^ = 0.55–0.78), while SVR was more effective for S and Mn (*R* ^2^ = 0.68–0.86) (Pullanagari et al., 2016). In oil palm leaf evaluations using multispectral images, RFR retrieved chlorophyll and Ca with *R* ^2^ = 0.75 and 0.71, respectively, whereas SVR predicted N with *R* ^2^ = 0.65 (Chungcharoen et al., 2022). A recent trend involves utilizing multiple models, for instance, a combination of PLSR and the Cubist model yielded excellent predictions for N, P, K, Mn, and Zn in cashew (Mahajan et al., 2024). A study using PCA and PLSR predicted traits associated with salinity stress for rice (Das et al., 2020a). In wheat, a study employed ML and eXplainable Artificial Intelligence to predict N status, achieving *R* ^2^ > 0.85 with RFR and Gradient Boosting models (H. Singh et al., 2022). Another study utilized a UAV platform with multispectral, RGB, and thermal infrared cameras to estimate the nitrogen nutrition index of wheat, achieving *R* ^2^ = 0.89 with GPR and improving model transferability by 11% using Transfer Component Analysis (Zhang et al., 2024). The superior performance of these models is attributed to their ability to capture complex, non-linear relationships between spectral features and nutrient levels (Ennaji et al., 2023; Zaji et al., 2022). However, the increased complexity of these sophisticated models poses challenges in fully interpreting their mechanisms (Molnar, 2020), and they require significant computational power and large datasets making them intractable for many circumstances (Jordan and Mitchell, 2015; Sze et al., 2017).

Wheat (*Triticum aestivum*) is one of the most important cereal crops grown for human food consumption providing approximately 15% of total calories, and is the most widely cultivated crop worldwide (Erenstein et al., 2022). It covers a significant portion of cultivated land spanning over 220 million hectares globally (Nduku et al., 2023). For roughly 36% of people worldwide, wheat is a key source of food (Khalid et al., 2023). The importance of wheat as a food crop has made ensuring steady production and enhancing wheat nutritional quality crucial goals for global food security and has motivated work to rapidly assess wheat plant health. This includes the estimation of nitrogen content (Bossung et al., 2022; Clay et al., 2012; Hansen and Schjoerring, 2003; Li et al., 2020), leaf area index (Das et al., 2020b; Jamali et al., 2023), biomass (Prasad et al., 2009; Sticksel et al., 2004), grain yield (Montesinos-López et al., 2017; Prasad et al., 2007; Thorp et al., 2017; Xie et al., 2020; Zhang et al., 2020), and sugars and starch (Robles-Zazueta et al., 2022). NIR spectroscopy has been utilized to assess nitrogen content and leaf mass per unit area in fresh and dried durum wheat plants (Ecarnot et al., 2013), as well as to evaluate leaf nitrogen content and leaf area index in field trials of wheat (Pimstein et al., 2007). This focus on nitrogen content and canopy structural traits was followed by a limited number of studies that address the ability of spectroscopy to quantify other nutrient contents, including phosphorus potassium, sulfur, calcium and magnesium (Ayala-Silva and Beyl, 2005; Mahajan et al., 2014; Pimstein et al., 2011; Yang et al., 2021). There has also been work using airborne spectroscopy (Raya-Sereno et al., 2021) and also work to evaluate bread wheat genotype responses to water stresses (Hernandez et al., 2015).

In this study, we focus on the examination of the use of leaf-level VSWIR spectroscopy observations to provide a high-throughput predictive capability for a wide range of winter wheat macro- and micronutrients. Using data collected over two growing seasons from breeding programs in central Ohio, we analyze data from 360 unique foliar samples for the following nutrient contents: nitrogen, phosphorus, potassium, calcium, magnesium, sulfur, boron, copper, iron, manganese, zinc, molybdenum, aluminum, and potassium, as well as moisture content. All fresh leaf samples had reflectance measurements conducted across the 350–2,500 nm range using a field spectrometer. This allowed us to evaluate correlations between foliar nutrient content variability and reflectance values, and further to evaluate the use of PLSR to model this broad range of nutrient contents. Three different PLSR component selection methods were contrasted to assess the trade-off between predictive performance and model complexity. Important regions of the spectrum for each foliar trait were identified using variable importance in projection (VIP) scores. Hence, the specific objectives of this paper were to (a) quantify and evaluate nutrient variability in winter wheat; (b) build quantitative models for predicting macronutrient and micronutrient concentrations from leaf reflectance using PLSR; (c) evaluate contrasting component selection methods for development of a set of final PLSR models; and (d) identify important regions of the VSWIR spectrum for the prediction of this wide range of foliar nutrients.

## 2 Materials and methods

### 2.1 Study area and leaf sampling

The experiments were conducted at two research sites situated at the Ohio Agricultural Research and Development Center (OARDC) of Ohio State University, Wooster, Ohio, USA (40°46′01.0″ N, 81°53′57.0″ W and 40°46′01.4″ N, 81°53′47.3″ W). Samples were collected during two crop growing cycles, in the spring of 2022 and 2023. Each site consisted of a winter wheat breeding population in which each genotype was planted in 1.5 × 3.0 m (4.6 m^2^)-sized plots. Utilizing this diverse breeding population allowed for maximizing the genetic influence on phenotypic traits (plant nutrient contents), making the breeding trials a potential source of the variability to enhance this dataset for model evaluation. Each wheat plot consisted of seven rows spaced 0.78 m apart and seeded with 100 g of seed. Nitrogen was applied in fall at 28 kg/ha and was supplemented in spring at 100 kg/ha. No irrigation or other fertilizer treatments were applied throughout the growing seasons.

Fresh leaf samples were collected from 360 wheat plots across the two study years: 180 plots in 2022 and 180 plots in 2023. Data acquisition (VSWIR leaf reflectance and leaf sampling for trait evaluation) was performed over multiple field visits (11 May 2022, 3 June 2022, 17 June 2022, 18 May 2023, 26 May 2023, and 1 June 2023), providing variability in plant growth stage, maturity, and environmental response. On each sampling date, 60 plots were randomly selected and sampled. For sample collection, multiple fully expanded mature leaves were harvested from each sampled plot and placed in labeled zip-lock bags in a cooler to prevent changes to leaf pigments and water loss due to transpiration. Immediately following the collection of leaves in the field, these leaves were transported to a lab where leaf spectroscopy measurements were immediately collected.

### 2.2 Hyperspectral reflectance data acquisition

Leaf spectroscopy measurements were collected using an ASD FieldSpec 4 Standard Resolution Spectroradiometer (Malvern Panalytical, Boulder, Colorado, USA). This instrument measures reflected radiance across the 350–2,500 nm wavelength range, which is then converted into reflectance by normalization against an observation made on a white reflectance target. Measurements are made using a 1.5-m-long fiber optic cable with a 25°field of view. Three distinct sensors in the instrument include a visible and near-infrared detector (VNIR) operating from 350 to 1,000 nm with 3-nm resolution, a shortwave infrared detector (SWIR 1) operating from 1,000 to 1,800 nm with 10-nm resolution, and another shortwave infrared detector (SWIR 2) covering 1,800 to 2,500 nm at 10-nm resolution (Malvern Panalytical, 2023). Following interpolation, the reflectance observations span 2,151 individual 1-nm wavebands that were used here for trait modeling.

Foliar reflectance spectra were collected using a leaf clip assembly with built-in contact probe, which allowed the fiber optic cable to view an illuminated portion of the leaf being sampled. The contact probe featured a 10-mm-diameter field of view. The leaf clip assembly used a halogen bulb to provide controlled illumination sufficient to capture leaf reflectance across the full spectral range examined here. The leaf clip assembly included a two-sided rotating head, with one side having a black panel and the other side having a white panel. Following a 30-min warm-up period for the instrument prior to data collection, two wheat leaves were positioned side by side and centered across the black background of the contact probe, covering the entire observation region of the probe (Danner et al., 2015). The leaf clip was then securely fastened to minimize measurement errors associated with stray light. Three independent readings were collected from the foliage in each sample bag, from which the spectra were averaged to ensure the representativeness of each foliage sample. White reference readings used to normalize each spectrum to reflectance values were recorded at 30-min intervals. This resulting dataset was subjected to splice correction and subsequently exported as ASCII text files using ViewSpec Pro Software (Analytical Spectral Devices Inc., Boulder, CO, USA) for further analysis. Once reflectance measurements had been completed for an entire set of leaf samples, they were then processed for tissue chemistry measurements.

### 2.3 Foliar chemistry and water content

Following the collection of reflectance measurements, the wet weight (*WW*, [g]) of each leaf sample was measured. The leaf samples were then placed inside labeled paper bags and dried in an oven maintained at approximately 50–60°C for several days until completely dried. Dry weight (*DW*, [g]) was then measured, allowing the moisture content (*MC*; [%]) of each sample to be calculated using the following formula (Morshedloo et al., 2016):


$$MC=\frac{WW-DW}{WW}\times 100$$

Plant tissue chemical analysis was conducted at the Service, Testing, and Research (STAR) laboratory at Ohio State University (Wooster, OH). Total nitrogen (N, [%]) in the samples was determined using Duma’s method (AOAC, 1970). The concentrations of the other plant elements analyzed here were quantified in micrograms per gram (µg/g) through a nitric acid microwave digestion system (Jones et al., 1991) and included phosphorus (P), potassium (K), calcium (Ca), magnesium (Mg), sulfur (S), aluminum (Al), boron (B), copper (Cu), iron (Fe), manganese (Mn), molybdenum (Mo), sodium (Na), and zinc (Zn). While all plants depend on these 14 nutrients for their overall growth and development, here we distinguish between macronutrients and micronutrients simply in the quantities required by the plant (Welch and Shuman, 1995).

### 2.4 Statistical modeling

PLSR was utilized to predict nutrient and water concentrations from the reflectance spectra (Ang and Seng, 2021; Wold et al., 2001). PLSR is effective for prediction problems with a large number of predictor variables relative to the number of observations, particularly when multi-collinearity exists across the predictor set (Geladi and Kowalski, 1986; Hubert and Branden, 2003; Sawatsky et al., 2015; Tobias, 2000). Here, we used the SIMPLS algorithm as implemented in the “*plsregress*” function of MATLAB to develop PLSR models of each leaf trait (de Jong, 1993). For each trait (nutrient contents and percent water content), a unique PLSR model development process was conducted using the wavelength range 450–2,400 nm. Wavelengths outside of this range were removed due to noise in reflectance values at each end of the spectra. We applied no transformations to reflectance data and nutrient distributions (Burnett et al., 2021). For each trait, models were developed using 1 to 30 components. For each model development iteration, the 360 data points were partitioned randomly into calibration (80% of data) and validation (remaining 20% of data) sets using a random variable generator in MATLAB. For each component trait and component number, this process of randomly splitting the data, calibrating the model, and evaluating the model on the validation data was performed 1,000 times to enhance the robustness of the analysis and mitigate potential biases stemming from random data splitting. Each of these 1,000 iterations additionally involved fivefold cross-validation within the calibration set (Fushiki, 2011). Predicted residual error sum of squares (PRESS), root mean squared error (RMSE), coefficient of determination (*R* ^2^), and the ratio of performance to deviation (RPD) served as measures of prediction accuracy.

In order to mitigate the risk of overfitting, it was imperative to carefully determine the optimal number of PLSR components (#Comp) (Alma, 2013). Here, we evaluated three distinct approaches for PLSR model selection to evaluate how each performed for this diverse set of wheat foliar traits when choosing the optimal number of PLSR components to retain in the final models.

#### 2.4.1 Minimum PRESS

The first approach involved identifying the number of components that produced the absolute minimum value of the PRESS statistic (PRESS_min_) for the validation fraction of the dataset. This approach has been widely used for model selection in vegetation spectroscopy (Acosta et al., 2023; Cherif et al., 2023; Oliveira and Santana, 2020; Stein et al., 2014; Wang et al., 2020; Yang et al., 2021).

#### 2.4.2 Backward penalty

We noted certain situations where the PRESS value for a reduced number of components only marginally exceeded the absolute PRESS_min_, such that simplifying the final model (i.e., reducing the number of components used in the model) would be possible with little negative impact on model performance. In such cases, a backward penalty approach was employed. Starting with the number of components defining PRESS_min_, models utilizing a successively reduced number of components were evaluated until the difference in the mean PRESS values for the validation fraction for consecutive numbers of components fell below a predefined threshold or penalty value (Tran et al., 2017). While the determination of this penalty value could be subjective, it might not always hold a statistical significance. To address this, we chose to use a relative threshold as a percentage (1.5%) of the maximum PRESS value from the validation set for each nutrient. This approach is referred to as “PRESS_adj_” below.

#### 2.4.3 Van der Voet statistic

The final approach evaluated here involved using randomization tests, known as permutation tests, which offer the advantage of utilizing the entire dataset in performance evaluation (Van der Voet, 1994). The Van der Voet statistic was used to randomly select various models and compare their residuals to those of the reference model that minimized PRESS. Our implementation involved a two-sided randomization *t*-test with a Van der Voet *T* ^2^ significance level of 1% (*p*-value < 0.01) to ascertain the dimensionality of the PLSR model. This approach is referred to as “Voet” below.

This exploration of component selection methods facilitated well-informed decisions about model complexity, to better ensure robust and reliable final models.

Finally, VIP scores were computed for the final model, serving as a metric to identify the regions of the reflectance spectrum that held significant importance in predicting leaf nutrient levels (Meacham et al., 2020; Nakaji et al., 2019). VIP scores are commonly employed in the variable selection process (Mehmood et al., 2012), reflecting the statistical significance of each independent variable (in this case, wavelengths) in the fitted PLSR model (Chong and Jun, 2005). A higher VIP score indicates a greater importance of the independent variable in explaining the variance of the dependent variable (in this case, nutrient and water concentrations) (Mehmood et al., 2020). Typically, a VIP score exceeding 1 serves as the criterion for selecting relevant variables (Akarachantachote et al., 2014).

## 3 Results and discussion

### 3.1 Nutrient concentration analysis

**Figure 1:**
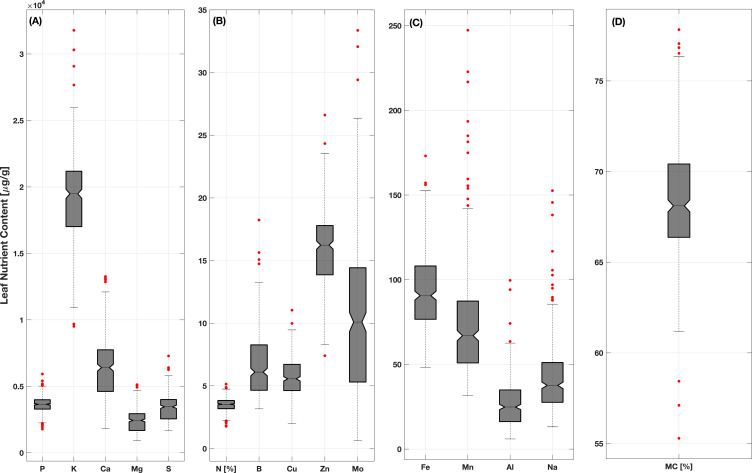
Box plots of wheat foliar trait data for the 360 samples analyzed in this study. Each box represents the interquartile range, which spans the middle 50% of the data. The lower and upper boundaries of the box correspond to the 25th (Q1) and 75th (Q2) percentiles, respectively. The horizontal center line in each box represents the sample median value. The dashed vertical lines indicate the region within 1.5 times the interquartile range, with red points showing specific data values beyond this range (i.e., outliers). The data in each panel groups traits with similar ranges of variability in [·g/g], with panel **(A)** presenting data for P, K, Ca, Mg and S; panel **(B)** presenting data for N (in %), B, Cu, Zn and Mo; panel **(C)** presenting data for Fe, Mn, Al and Na; and panel **(D)** presenting data for MC (in %).

Descriptive statistics of the observed foliar traits are presented in **Table 1** and visually depicted as box plots in **Figure 1**. Laboratory analysis showed that the concentrations of all nutrients examined here exhibited a wide range with large standard deviations. Mo varied greatly with 61.19% CV whereas MC varied the least, having only 5.41% CV. The mean element concentrations were 3.47%, 3,635.34 µg/g, 19,179.17 µg/g, 6,210.99 µg/g, 2,409.48 µg/g, 3,379.64 µg/g, 6.60 µg/g, 5.66 µg/g, 93.17 µg/g, 73.70 µg/g, 15.78 µg/g, 10.39 µg/g, 26.86 µg/g, 41.55 µg/g, and 68.57% for N, P, K, Ca, Mg, S, B, Cu, Fe, Mn, Zn, Mo, Al, Na, and MC, respectively. Notably, none of the elements exhibited a highly skewed distribution as evident in the histograms in **Supplementary Figure S1A**. Furthermore, the analysis revealed that P, K, B, Cu, and Fe exhibited nearly uniform distributions for the sampling performed here.

**Table 1:** Statistics describing observed leaf trait values of winter wheat samples examined in this study.

Statistics	Min	Max	Mean	Median	SD	CV (%)
**N [%]**	1.77	5.14	3.47	3.53	0.59	16.96
**P [µg/g]**	1,783.00	5,926.00	3,635.34	3,645.00	660.74	18.18
**K [µg/g]**	9,511.00	31,780.00	19,179.17	19,480.00	3,146.51	16.41
**Ca [µg/g]**	1,839.00	13,260.00	6,210.99	6,405.50	2,287.14	36.82
**Mg [µg/g]**	918.10	5,123.00	2,409.48	2,435.50	846.76	35.14
**S [µg/g]**	1,681.00	7,286.00	3,379.64	3,459.50	990.40	29.30
**B [µg/g]**	3.17	18.23	6.60	6.10	2.52	38.13
**Cu [µg/g]**	1.98	11.04	5.66	5.56	1.52	26.79
**Fe [µg/g]**	48.32	173.10	93.17	90.62	23.60	25.33
**Mn [µg/g]**	31.54	247.30	73.70	66.97	32.10	43.56
**Zn [µg/g]**	7.41	26.61	15.78	16.23	3.34	21.15
**Mo [µg/g]**	0.63	33.37	10.39	10.10	6.36	61.19
**Al [µg/g]**	5.96	99.63	26.86	24.82	13.92	51.81
**Na [µg/g]**	13.11	152.60	41.55	37.62	20.50	49.34
**MC [%]**	55.29	77.83	68.57	68.11	3.71	5.41

These statistics summarize the full set of 360 leaf samples. Presented here are the minimum (Min) and maximum (Max) values of each trait, along with the mean, median, standard deviation (SD), and the coefficient of variation (CV) expressed as a percentage (SD/Mean × 100).

**Figure 2:**
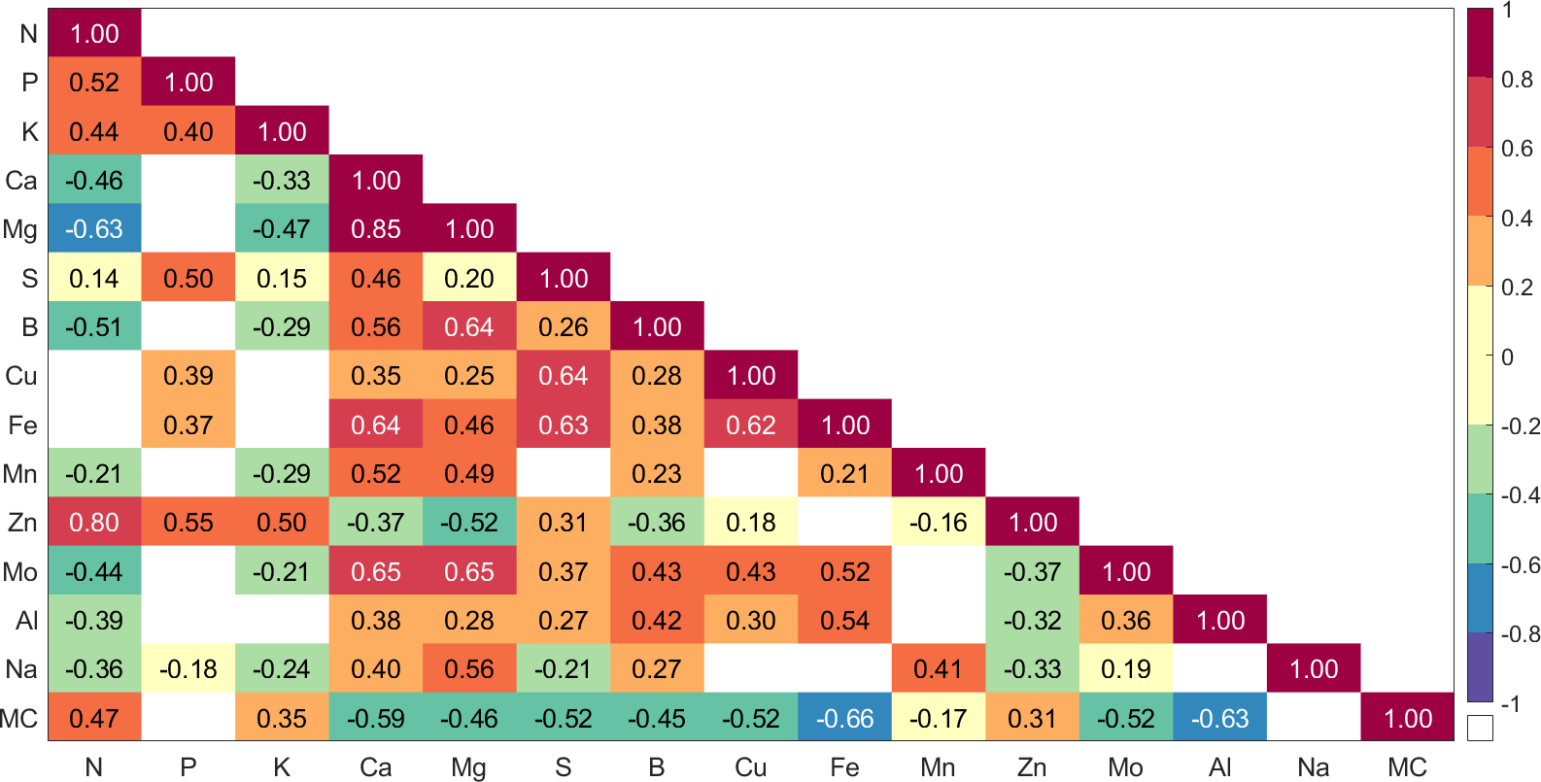
Heatmap illustrating the cross-correlations among the 14 leaf nutrients and moisture content. Each square represents the Pearson correlation coefficient between two leaf traits, with color intensity indicating the strength and direction of the correlation. A significance threshold of *p* < 0.01 was used, with non-significant correlations (NS*) shown as white squares.

A heatmap of the correlation coefficients (*r*) between each of the leaf traits is presented in **Figure 2**. The majority of the correlations were significant at the 0.01 probability level (non-significant values are displayed in white). The highest correlation between any two nutrients was for Ca and Mg with a correlation of 0.85, with the correlation between N and Zn also quite high at 0.80. Fe had strong positive correlations (*r* > 0.6) with three other nutrients: Ca, S, and Cu. Mo had strong positive correlations (*r* > 0.6) with two other nutrients: Ca and Mg. The strongest negative correlations were between N and Mg (*r* = −0.63), and between water content and Fe (*r* = −0.66) and Al (*r* = −0.63). Of all 105 pairs of traits, 21 (20%) were not significant, 53 (50.5%) were positively correlated, and 31 (29.5%) were negatively correlated.

### 3.2 Spectral and correlation analysis

**Figure 3:**
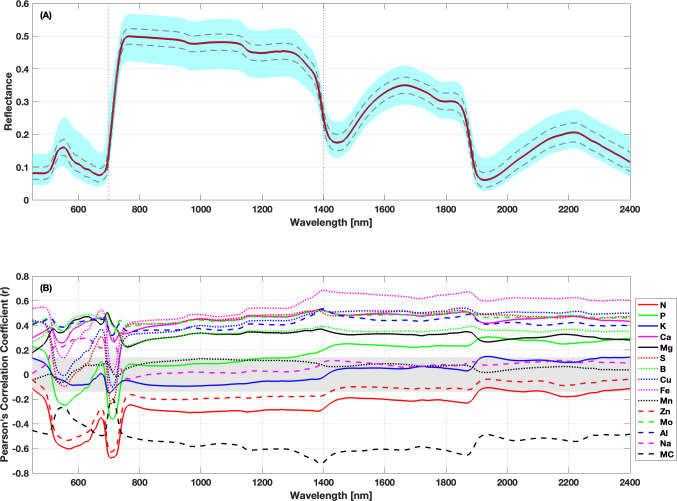
Leaf reflectance spectra and correlations with foliar traits across the reflectance range. **(A)** displays the mean (red solid line) leaf reflectance spectrum across the 450–2,400 nm wavelength range, along with one standard deviation bands (dashed gray lines) and the full reflectance range observed here (blue-shaded region). **(B)** presents the Pearson correlation coefficients between nutrient concentrations and leaf reflectance for each wavelength. The gray shaded regions represent correlation values that are not significant at the 1% significance level.

The mean reflectance spectrum of the full collection of wheat leaf samples analyzed here (**Figure 3A**) exhibits the typical structure characteristic of healthy green foliage (Hunt et al., 2015). While the reflectance patterns across the wheat foliage spanning different genotypes and growth stages are quite similar, subtle variations at specific wavelengths within the VIS, NIR, and SWIR regions hold the potential to predict leaf macronutrient and micronutrient contents. The variability in the spectra is greatest in VIS, NIR, and SWIR regions and lowest in the red edge (650–750 nm) and two water absorption bands (1,350–1,420 and 1,680–1,700 nm). **Supplementary Figure S1B** displays the average leaf reflectance spectra for the six sampling dates spanning May 2022 to June 2023. A noticeable trend is observed across the visible, NIR, and SWIR regions, where reflectance is lower during the earlier dates (May) and gradually increases in the later dates (June). This trend may suggest seasonal variations in chlorophyll concentration, water content, and internal leaf structure.

To identify the strength of the relationships between nutrients and specific wavelength regions, the foliar traits were correlated with the reflectance data at each wavelength across the spectral range used here. These correlations are shown in **Figure 3B**. There were many significant correlations between trait values and the leaf reflectance at the 1% significance level (α = 0.01). Correlation values ranged from −0.73 to 0.69. Ca, Mg, S, B, Cu, Fe, and Al displayed consistent positive correlations across the spectral range. N, Zn, and MC exhibited consistently negative correlations across the full spectral range, with Zn correlations becoming statistically insignificant at wavelengths longer than 1,400 nm. We also observe that foliar traits exhibiting higher and positive *r* values in **Figure 2** demonstrate a congruent impact on leaf reflectance. For instance, the Ca–Mg pair (*r* = 0.85) displays positive correlations with reflectance. The Mo–Mg pair (*r* = 0.65) shows a similar positive trend, and the Zn–N pair (*r* = 0.80) exhibits a negative association with reflectance. Conversely, traits with more negative *r* values tend to exhibit divergent effects on reflectance. Notably, MC demonstrates a negative correlation with reflectance, while Fe displays a positive correlation, resulting in an *r* value of −0.66. Similar patterns emerge for the MC–Al and Mg–N pairs. K, Mn, and Na had insignificant correlations across most of the spectral range. Overall, Fe and MC had the largest positive and negative correlations at wavelengths longer than the visible range, respectively.

Multiple studies in the literature have examined leaf-level correlations between nutrient contents and VSWIR reflectance. Stein et al. (2014) observed negative correlations between Ca and Mg concentrations in the VIS and NIR spectral ranges (450–1,500 nm) for loblolly pine. They also reported positive Spearman rank correlation coefficients for elements such as N, P, and K. In contrast, our analysis reveals that N and K concentrations are negatively correlated in the VIS and NIR region, while P, Ca, and Mg show positive correlations. Our findings align with those of Yoder and Pettigrew-Crosby (1995), who observed a negative correlation in the N content of maple leaves within the 500–1,500 nm range. The findings of Zhai et al. (2012) also mirrored our results for the correlation patterns of P and K content, which fluctuated closely around zero. Additionally, Oliveira et al. (2019) identified positive correlation values for B, Mn, Ca, Fe, and Mg in Eucalyptus trees within the 400–730 nm range, consistent with our results. K displayed a negative correlation in the 400–730 nm range (Oliveira et al., 2019), transitioning to a positive correlation beyond 730 nm wavelength, akin to our observations of K, which exhibited a shift from negative to positive correlation at approximately 1,400 nm. A similar inflection point for correlation change was identified at approximately 700 nm (Pimstein et al., 2011), while a comparable shift at approximately 840 nm was seen for cowpea (Amaral et al., 2022). In the case of P content in wheat crops, Pimstein et al. (2011) found positive correlations in the NIR and SWIR regions (>1,100 nm), consistent with our results. Our findings indicated a negative correlation between N content with leaf spectra in wheat, a relationship also previously observed (Mahajan et al., 2014). Similar to our analysis, Amaral et al. (2022) identified a positive correlation for Ca and a negative correlation for Zn in the visible range, albeit for cowpeas. Our moisture content correlations aligns with similar peaks reported (Ng et al., 2007). Kovar et al. (2019) examined the relationship between leaf water content and hyperspectral reflectance in soybeans and found that the correlation between moisture content and spectral data fluctuated around zero, in contrast to the strongly negative correlations between MC and reflectance that we found. Overall, the data shows substantial correlations between reflectance and nutrient and water content across this diverse wheat dataset, motivating the examination of VSWIR spectroscopy to model these traits in wheat.

### 3.3 Model performance

The performance of the optimal PLSR models using the three different model selection techniques is presented in **Table 2**. The table presents normalized PRESS and RMSE values. Normalized PRESS is obtained by dividing the PRESS value of either the calibration or validation set by the maximum PRESS value within that respective set. RMSE is expressed as a percentage of the mean value of that trait across the full dataset. PLSR models for nutrients differed greatly in their predictive capabilities when using different selection methods. First, we examine the nitrogen models to illustrate the trade-offs between model performance metrics and model complexity. PRESS_min_ yields the highest *R* ^2^ and RPD (0.86 and 2.68 respectively) and lowest error (PRESS = 0.16, RMSE = 6.33) in the validation set, which are typically considered indicators of good model performance. However, despite these seemingly favorable outcomes, PRESS_min_ retains a very high number of components (19), potentially resulting in a model with higher complexity that may model elements of noise in the dataset. This complexity can introduce unwanted noise into the model, as observed in the PLSR coefficient plots (**Supplementary Figure S2C**). The Voet method falls short for similar reasons as it does manage to mitigate the use of some excessive components but residual noise persists in the model. In contrast, the PRESS_adj_ method demonstrates strong predictive performance with an *R* ^2^ of 0.84, an RPD of 2.45, and a slightly higher error (PRESS = 0.19, RMSE = 6.91) using fewer components (14), thereby resulting in smoother model coefficients. Similar observations can be made for the models of K, Ca, Mg, S, Cu, Zn, Mo, and MC. It is crucial to strike a balance between accuracy, error, and model simplicity, as excessively complex models may introduce noise and compromise interpretability and extensibility to other datasets and broader applicability. Here, we demonstrate that the success of the PRESS_adj_ method, compared to the widely used PRESS_min_ method and the less commonly employed Voet method, lies in its ability to retain an optimal number of components while explaining a comparable amount of nutrient variation to other selection methods that tend to retain a higher number of components. A detailed comparison of the three model selection methods is presented for all foliar traits in **Supplementary Figures S2** through S16.

**Table 2:** Summary details of leaf trait prediction models for the three selection methods evaluated here.

Nutrient	Selection method	# Comp	*R* ^2^		RPD		Normalized PRESS		RMSE [%]	
			Cal	Val	Cal	Val	Cal	Val	Cal	Val
**N [%]**	PRESS_min_	19	0.89	0.86	3.10	2.68	0.12	0.16	5.47	6.33
	PRESS_adj_	14	0.87	0.84	2.80	2.45	0.15	0.19	6.05	6.91
	Voet	16	0.88	0.85	2.94	2.56	0.14	0.17	5.76	6.62
**P [µg/g]**	PRESS_min_	20	0.57	0.42	1.53	1.30	0.47	0.65	11.85	13.99
	PRESS_adj_	20	0.57	0.42	1.53	1.30	0.47	0.65	11.85	13.99
	Voet	16	0.54	0.39	1.48	1.26	0.5	0.7	12.26	14.45
**K [µg/g]**	PRESS_min_	26	0.72	0.55	1.87	1.49	0.3	0.46	8.75	11.03
	PRESS_adj_	18	0.64	0.53	1.67	1.45	0.38	0.49	9.84	11.35
	Voet	23	0.68	0.56	1.78	1.48	0.33	0.46	9.21	11.08
**Ca [µg/g]**	PRESS_min_	30	0.88	0.76	2.85	2.01	0.17	0.34	12.9	18.35
	PRESS_adj_	26	0.85	0.75	2.55	1.97	0.21	0.34	14.45	18.67
	Voet	29	0.87	0.75	2.75	2.00	0.18	0.34	13.38	18.45
**Mg [µg/g]**	PRESS_min_	29	0.9	0.82	3.24	2.33	0.11	0.21	10.86	15.1
	PRESS_adj_	21	0.85	0.78	2.57	2.13	0.18	0.25	13.69	16.49
	Voet	27	0.89	0.81	3.12	2.32	0.13	0.22	11.54	15.41
**S [µg/g]**	PRESS_min_	28	0.81	0.69	2.32	1.77	0.26	0.44	12.61	16.53
	PRESS_adj_	18	0.73	0.66	1.93	1.71	0.37	0.47	15.19	17.13
	Voet	26	0.8	0.68	2.22	1.74	0.28	0.46	13.17	16.81
**B [µg/g]**	PRESS_min_	14	0.53	0.46	1.47	1.34	0.56	0.67	25.92	28.35
	PRESS_adj_	13	0.53	0.44	1.45	1.34	0.57	0.68	26.23	28.5
	Voet	11	0.49	0.42	1.41	1.30	0.61	0.71	27.14	29.26
**Cu [µg/g]**	PRESS_min_	16	0.62	0.53	1.63	1.44	0.52	0.64	16.44	18.56
	PRESS_adj_	10	0.57	0.52	1.52	1.44	0.59	0.65	17.68	18.56
	Voet	13	0.59	0.53	1.55	1.44	0.56	0.65	17.15	18.56
**Fe [µg/g]**	PRESS_min_	16	0.74	0.69	1.98	1.78	0.44	0.52	12.82	14.22
	PRESS_adj_	13	0.73	0.68	1.91	1.75	0.47	0.54	13.23	14.51
	Voet	12	0.71	0.66	1.87	1.72	0.49	0.56	13.52	14.75
**Mn [µg/g]**	PRESS_min_	18	0.53	0.41	1.47	1.28	0.47	0.61	29.66	33.98
	PRESS_adj_	18	0.53	0.41	1.47	1.28	0.47	0.61	29.66	33.98
	Voet	14	0.48	0.38	1.38	1.24	0.53	0.64	31.29	34.83
**Zn [µg/g]**	PRESS_min_	26	0.81	0.7	2.30	1.82	0.21	0.33	9.19	11.6
	PRESS_adj_	13	0.72	0.66	1.89	1.72	0.31	0.38	11.22	12.29
	Voet	24	0.8	0.69	2.23	1.79	0.23	0.35	9.51	11.79
**Mo [µg/g]**	PRESS_min_	24	0.67	0.51	1.75	1.40	0.45	0.69	34.95	43.71
	PRESS_adj_	20	0.63	0.48	1.65	1.37	0.51	0.72	37.06	44.67
	Voet	21	0.64	0.49	1.68	1.37	0.49	0.72	36.49	44.57
**Al [µg/g]**	PRESS_min_	22	0.64	0.53	1.66	1.45	0.47	0.63	31.2	35.74
	PRESS_adj_	21	0.63	0.53	1.64	1.44	0.49	0.63	31.65	35.97
	Voet	18	0.61	0.49	1.61	1.41	0.51	0.68	32.47	37.38
**Na [µg/g]**	PRESS_min_	15	0.53	0.45	1.46	1.34	0.49	0.58	33.89	36.95
	PRESS_adj_	13	0.5	0.43	1.42	1.32	0.52	0.59	34.83	37.31
	Voet	11	0.45	0.41	1.36	1.28	0.56	0.63	36.35	38.42
**MC [%]**	PRESS_min_	16	0.89	0.86	3.09	2.67	0.17	0.23	1.75	2.03
	PRESS_adj_	9	0.85	0.84	2.63	2.46	0.24	0.27	2.06	2.2
	Voet	13	0.88	0.85	2.92	2.61	0.19	0.24	1.85	2.07

Displayed here for each trait and selection method are the optimal number of components of the final models (# Comp), the coefficient of determination (R^2^), the ratio of performance to deviation (RPD), the normalized predicted residual error sum of squares (PRESS) statistic, and the root mean square error (RMSE [%]) for both calibration (Cal) and validation (Val) datasets.

**Figure 4:**
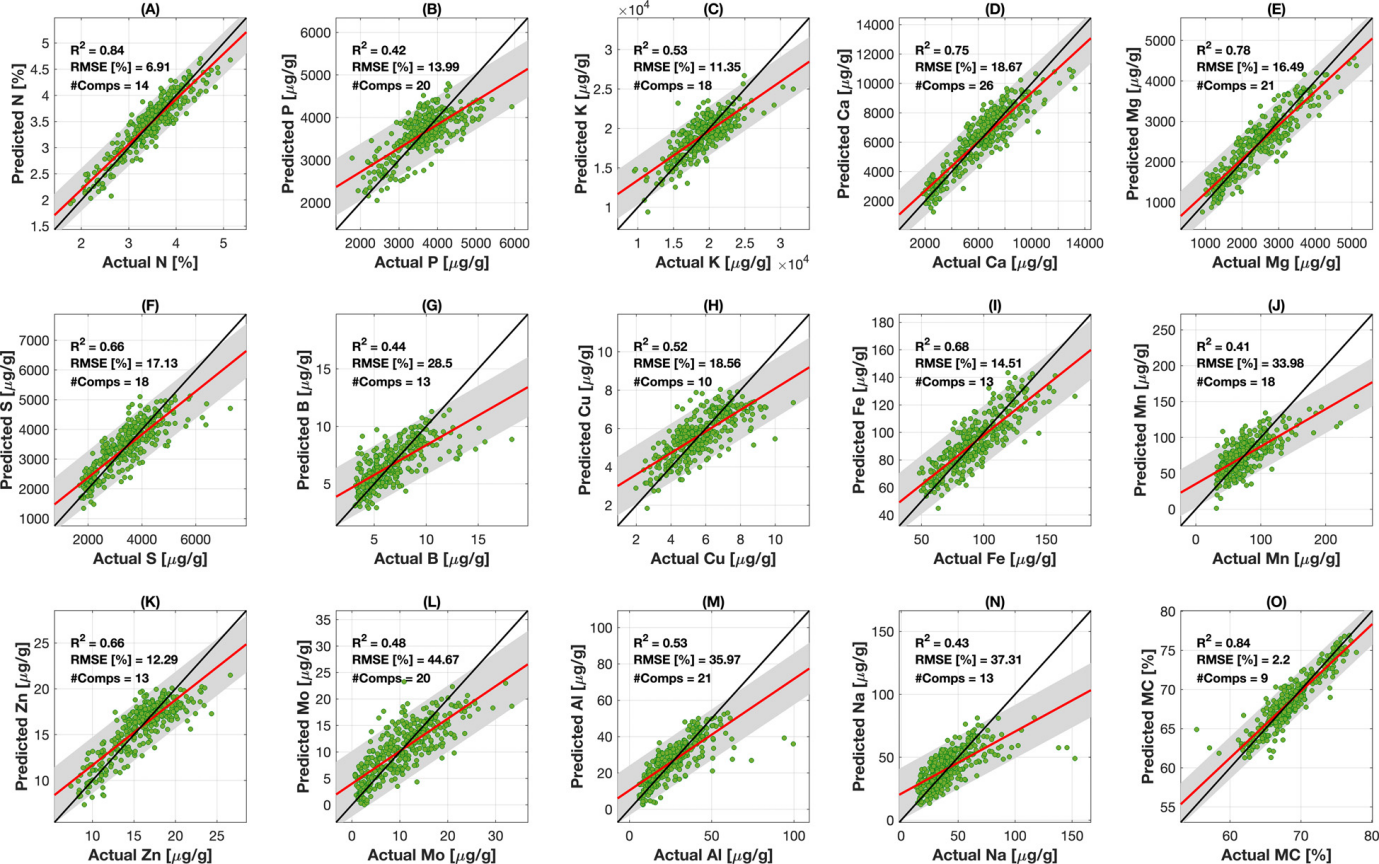
PLSR model performance for the 15 wheat foliar traits using PRESS_adj_ for model selection. Each panel shows a comparison between predicted (vertical axes) and observed (horizontal axes) trait values. In each panel, the black solid line is the 1-1 line, the red line shows the best linear fit line between the model and observed data, the gray shaded region denotes 95% confidence bounds around the fit line serving as the measure of precision of the prediction, with the green dots representing individual data points. For each trait, the coefficient of determination (*R* ^2^), the root mean square error (RMSE) for the validation fraction, and number of components are presented. Parts **A–O** correspond to the model performance for N, P, K, Ca, Mg, S, B, Cu, Fe, Mn, Zn, Mo, Al, Na, MC, respectively.

The performance of the final PLSR regression models using PRESS_adj_ for model selection is presented in **Figure 4**. This figure demonstrates the broadly strong model performance that PLSR using PRESS_adj_ demonstrates for a wide range of macro- and micronutrients and foliar water content. The best-performing models are associated with N (*R* ^2^ = 0.84, RPD = 2.45, RMSE = 6.91), MC (*R* ^2^ = 0.84, RPD = 2.46, RMSE = 2.20), Mg (*R* ^2^ = 0.78, RPD = 2.13, RMSE = 16.49), and Ca (*R* ^2^ = 0.75, RPD = 1.97, RMSE = 18.67). Other nutrients for which PLSR models showed intermediate to high predictive accuracy were Fe (*R* ^2^ = 0.68, RPD = 1.75, RMSE = 14.51), Zn (*R* ^2^ = 0.66, RPD = 1.72, RMSE = 12.29), and S (*R* ^2^ = 0.66, RPD = 1.71, RMSE = 17.13). In contrast, the models for K (*R* ^2^ = 0.53, RPD = 1.45, RMSE = 11.35), Al (*R* ^2^ = 0.53, RPD = 1.44, RMSE = 35.97), Cu (*R* ^2^ = 0.52, RPD = 1.44, RMSE = 18.56), Mo (*R* ^2^ = 0.48, RPD = 1.37, RMSE = 44.67), B (*R* ^2^ = 0.44, RPD = 1.34, RMSE = 28.50), Na (*R* ^2^ = 0.43, RPD = 1.32, RMSE = 37.31), P (*R* ^2^ = 0.42, RPD = 1.30, RMSE = 13.99), and Mn (*R* ^2^ = 0.41, RPD = 1.28, RMSE = 33.98) exhibit a moderate level of agreement with observed concentrations. In the work by Chang et al. (2001), three distinct categories were established to evaluate model reliability based on the RPD values. Models with an RPD greater than 2.0 are considered excellent, those with an RPD between 1.40 and 2.00 are classified as fair, while models with an RPD below 1.40 are deemed non-reliable. In our analysis, the majority of the nutrient models were classified as either excellent or fair, with the exceptions being Mo, B, Na, P, and Mn. In the vast majority of cases, the predicted values fall within the 95% confidence interval region, as illustrated in **Figure 4**.

**Figure 5:**
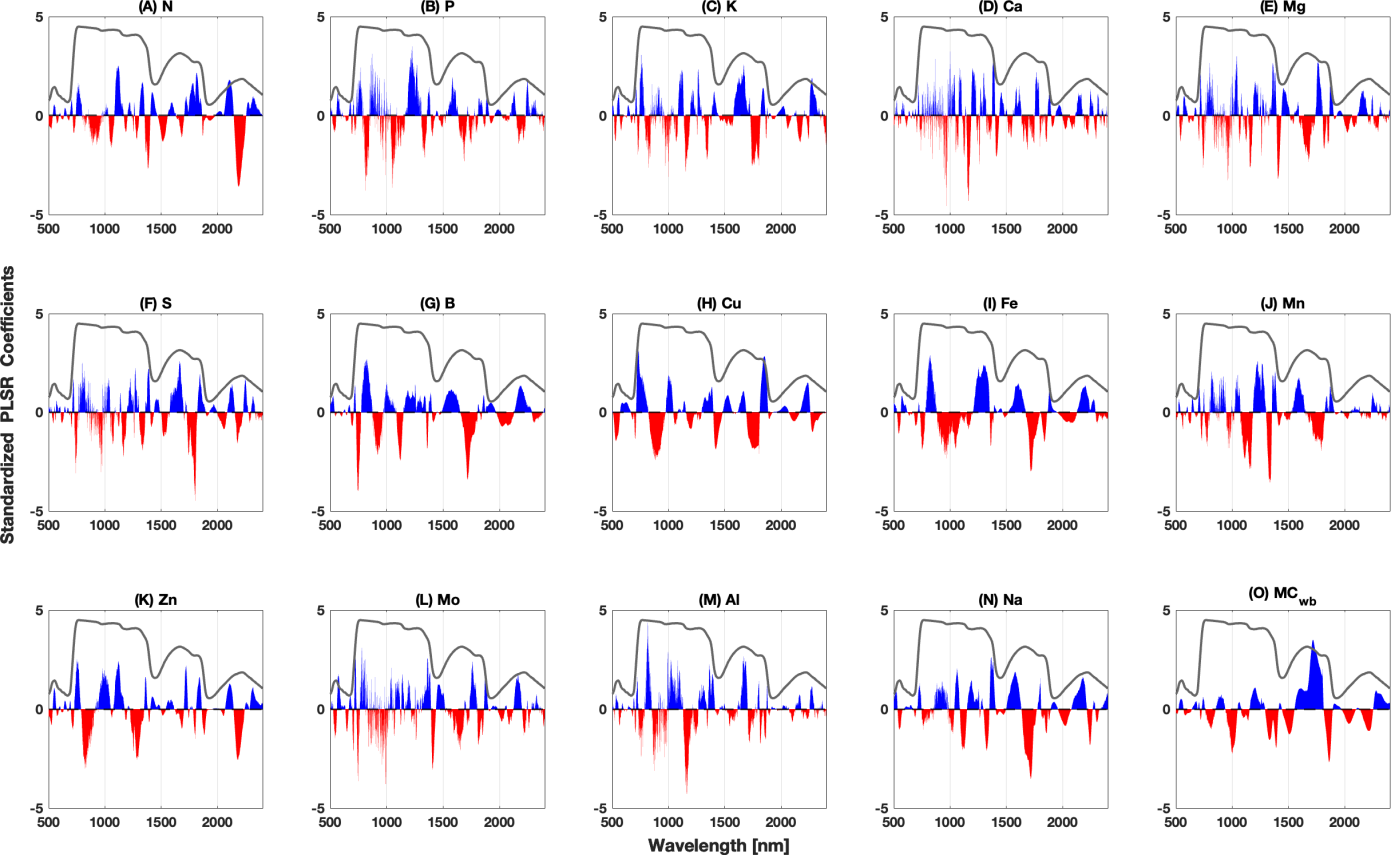
Standardized PLSR coefficient plots spanning the wavelength range from 450 to 2,400 nm using PRESS_adj_ for model selection. Blue regions indicate positive coefficient values while red hues represent negative coefficient values. The gray line shows the mean reflectance spectrum (multiplied by nine for display purposes) for reference. The coefficients presented here have been standardized to have a mean of zero and a standard deviation of one. Parts **A–O** correspond to the model coefficients for N, P, K, Ca, Mg, S, B, Cu, Fe, Mn, Zn, Mo, Al, Na, MC, respectively.

The PLSR coefficients across all the wavelengths produced by the PRESS_adj_ method are shown in **Figure 5**. The magnitudes of the regression coefficients serve as indicators of the impact of individual wavelengths and wavelength ranges on model predictions. These coefficients denote the weights assigned to each predictor variable in the model, with larger coefficients suggesting a stronger association between predictors and predictands (Sawatsky et al., 2015). The diverse set of nutrients examined here exhibited peaks in distinct wavelength regions that spanned the entire spectrum. The sign of each coefficient reveals the direction of the relationship between the predictors and predictands, with a positive coefficient signifying a positive relationship (as the predictor variable increases, the response variable tends to increase) and a negative coefficient signifying a negative relationship (as the predictor variable increases, the response variable tends to decrease).

**Figure 6:**
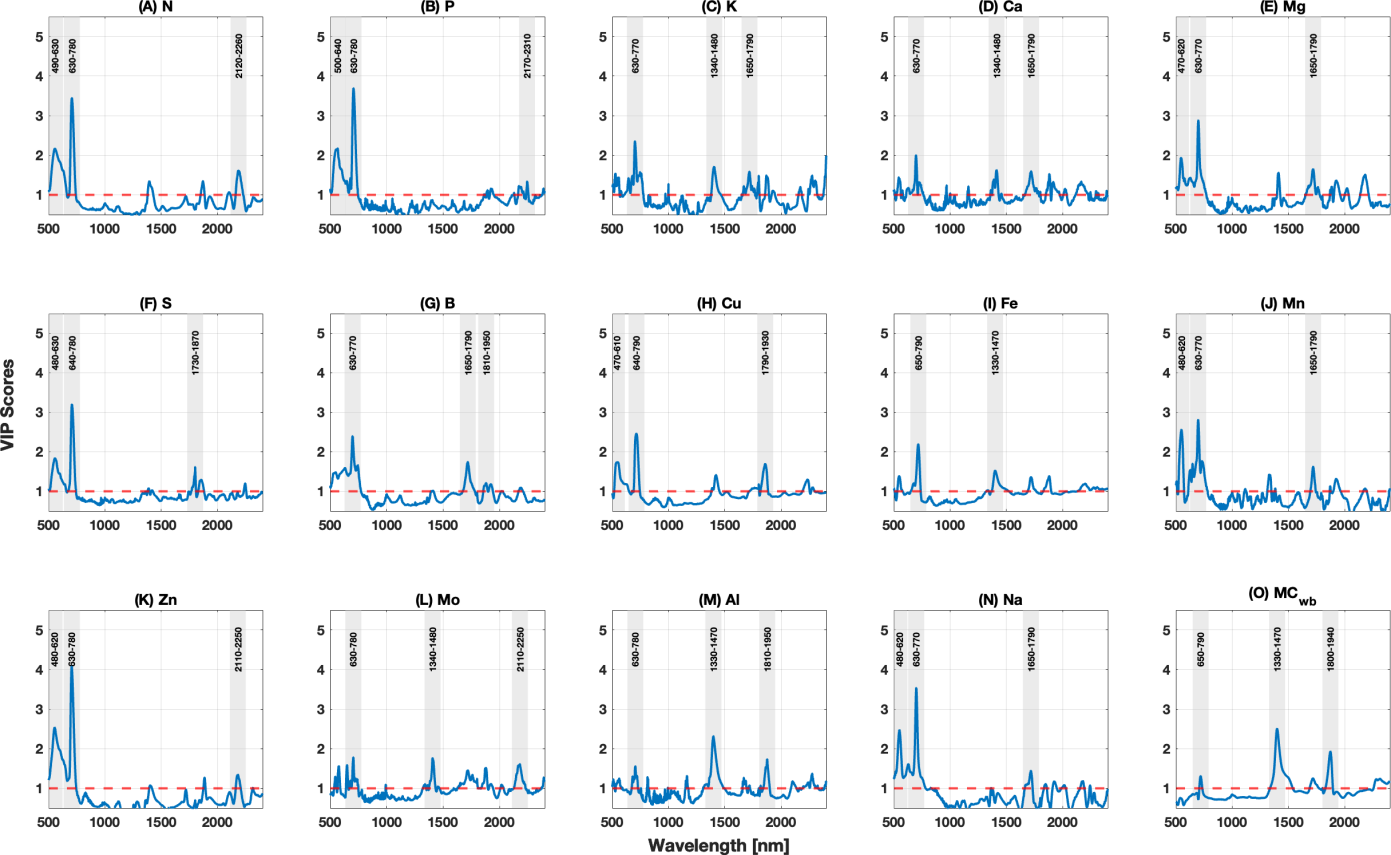
Variable Importance in Projection (VIP) scores across the full spectral range used for PLSR modeling. Solid blue lines are the VIP scores, with the threshold value of 1 shown as a red dashed line. The shaded gray regions correspond to the areas surrounding the top three peaks in the VIP scores, indicative of regions of the spectrum most important for modeling each leaf trait. Parts **A–O** correspond to the VIP scores for N, P, K, Ca, Mg, S, B, Cu, Fe, Mn, Zn, Mo, Al, Na, MC, respectively.

An associated method to assess important regions of the spectrum is the evaluation of VIP scores across the spectrum (**Figure 6**). Here, we utilize a VIP threshold value of 1 to indicate those spectral regions important to model predictions (Mehmood et al., 2020). All wavelengths with VIP scores above this value are considered significant in the final model prediction. In **Figure 6**, we highlight the wavelength ranges that represent the three highest peak values in VIP scores. These peaks usually align with wavelengths where essential physiological or biochemical processes in leaves affect reflectance (Curran, 1989). For almost all nutrients, the highest peak of VIP scores comes in the region of the spectrum spanning the red and red-edge wavelengths (630–770 nm) and the green region (550–560 nm) of the spectrum, as this region is associated with chlorophyll absorption crucial for photosynthesis. P, S, Zn, and Na showed the highest VIP scores (>3) in the 630–780 nm region. Generally, the VIP scores fall below the threshold across the NIR region for most nutrients. In this region, peaks are often linked to leaf internal structure and water content, which significantly affect light scattering within the leaf. The next highest peaks for most traits come in the SWIR region (1,300–2,400 nm), where they often correspond to water absorption bands and the presence of organic compounds, which are important for assessing leaf water status and stress indicators. For MC, the highest two peaks come in the SWIR region where there is greater sensitivity of reflectance to water content. These locations of important VIP scores and higher magnitude PLSR coefficients occurring in the visible and NIR regions are in agreement with studies that deal with other plant species such as pinot noir (Lyu et al., 2023), eucalyptus (Oliveira and Santana, 2020), temperate and boreal tree species (Serbin et al., 2014), and citrus (Acosta et al., 2023).

## 4 Conclusions

This study demonstrates the potential of using VSWIR spectroscopy as a rapid and non-destructive sensing technique, along with PLSR, to quantify the contents of 14 macro- and micronutrients in green winter wheat foliage, as well as foliar water content. Our sampling of winter wheat foliage at different growth stages over two field seasons demonstrated the wide range of variability in these foliar traits despite no differences in treatment. For most traits, there were extensive regions of the spectrum examined here (350–2,500 nm) for which there were significant correlations between trait values and reflectance, highlighting the potential of VSWIR sensing to capture subtle variations in even micronutrients with low concentrations.

This study provided a comprehensive evaluation of a wide range of macro- and micronutrients crucial for winter wheat growth, providing a wider lens on nutrient content variability than is typically provided in studies focused on one or a few macronutrients. This allowed us to evaluate the ability of VSWIR reflectance and PLSR to capture trait variability, particularly for some poorly studied micronutrients that pose challenges to model retrievals due to low concentrations in green foliar tissue. The models we developed exhibited good to excellent predictive performance across this wide range of foliar traits. The evaluation of model coefficient variability and VIP scores pointed to critical regions of the spectrum most important for predicting each trait. This analysis has the potential to guide sensor development focused on the quantification of a subset of foliar nutrients with simplified sensors that cover only the most critical spectral regions.

Our methodology provided a systematic comparison between three PLSR component selection methods. These included the widely used method of evaluation of the absolute minimum value of the PRESS statistic, as well as the less widely adopted Van der Voet’s two-sided randomization *t*-test, and a backward iteration process developed here to minimize model complexity while retaining sufficient predictive performance. Our analysis revealed that the backward iteration method struck an ideal balance between model complexity and predictive accuracy. In general, this method resulted in fewer components retained in the final model relative to the other two methods, providing confidence that overfitting had been reduced, making model predictions more reliable and providing a path to further evaluation of heuristic methods to reduce overfitting in PLSR model development.

This work has the potential to significantly improve precision wheat management by demonstrating the potential of spectroscopic sensors to provide accurate evaluations of foliar nutrient contents, allowing managers to make decisions informed broadly on nutrient health. Likewise, accurate and rapid assessments of foliar nutrient profiles have the potential to impact breeding decisions, potentially improving selection and accelerating the breeding cycle. In both cases, this study points to the potential for this technology to be applied to other agricultural systems.

## Data Availability

The raw data supporting the conclusions of this article will be made available by the authors, without undue reservation.

## References

[B1] Acosta M. Quiñones A. Munera S. de Paz J. M. Blasco J. (2023). Rapid prediction of nutrient concentration in citrus leaves using vis-NIR spectroscopy. Sensors 23, 6530. doi: 10.3390/s23146530 37514824 PMC10386652

[B2] Adams M. L. Norvell W. A. Philpot W. D. Peverly J. H. (2000). Spectral detection of micronutrient deficiency in ‘Bragg’ Soybean. Agron. J. 92, 261–268. doi: 10.1007/s100870050031

[B3] Ahmed N. Zhang B. Bozdar B. Chachar S. Rai M. Li J.. (2023). The power of magnesium: unlocking the potential for increased yield, quality, and stress tolerance of horticultural crops. Front. Plant Sci. 14. doi: 10.3389/fpls.2023.1285512 PMC10628537 37941670

[B4] Akarachantachote N. Chadcham S. Saithanu K. (2014). Cutoff threshold of variable importance in projection for variable selection. Int. J. Pure Apllied Mathematics 94, 307–322. doi: 10.12732/ijpam.v94i3.2

[B5] Alma Ö.G. (2013). Performance comparisons of model selection criteria: AIC, BIC, ICOMP and Wold’S for PLSR. J. Stat. Res. 10, 15–34.

[B6] Amaral J. B. C. Lopes F. B. de Magalhães A. C. M. Kujawa S. Taniguchi C. A. K. dos Santos Teixeira A.. (2022). Quantifying nutrient content in the leaves of cowpea using remote sensing. Appl. Sci. 12, 458. doi: 10.3390/app12010458

[B7] Ang K. L.-M. Seng J. K. P. (2021). Big data and machine learning with hyperspectral information in agriculture. IEEE Access 9, 36699–36718. doi: 10.1109/ACCESS.2021.3051196

[B8] AOAC (1970). Official methods of analysis. Arlington, Virginia, USA: Association of Official Analytical Chemists, Inc.

[B9] Araus J. L. Cairns J. E. (2014). Field high-throughput phenotyping: the new crop breeding frontier. Trends Plant Sci. 19, 52–61. doi: 10.1016/j.tplants.2013.09.008 24139902

[B10] Asner G. (2008). “Hyperspectral Remote Sensing of Canopy Chemistry, Physiology, and Biodiversity in Tropical Rainforests,” in Hyperspectral Remote Sensing of Tropical and Sub-Tropical Forests (Boca Raton, FL, USA: Taylor & Francis Group LLC.), 261–296. doi: 10.1201/9781420053432.ch12

[B11] Asner G. P. Brodrick P. G. Anderson C. B. Vaughn N. Knapp D. E. Martin R. E. (2016). Progressive forest canopy water loss during the 2012–2015 California drought. Sustainability Sci. 113, E249–E255. doi: 10.1073/pnas.1523397113 PMC4720336 26712020

[B12] Asner G. P. Martin R. E. Carranza-Jiménez L. Sinca F. Tupayachi R. Anderson C. B.. (2014). Functional and biological diversity of foliar spectra in tree canopies throughout the Andes to Amazon region. New Phytol. 204, 127–139. doi: 10.1111/nph.12895 24942328

[B13] Ayala-Silva T. Beyl C. A. (2005). Changes in spectral reflectance of wheat leaves in response to specific macronutrient deficiency. Adv. Space Res. 35, 305–317. doi: 10.1016/j.asr.2004.09.008 15934211

[B14] Azadnia R. Rajabipour A. Jamshidi B. Omid M. (2023). New approach for rapid estimation of leaf nitrogen, phosphorus, and potassium contents in apple-trees using Vis/NIR spectroscopy based on wavelength selection coupled with machine learning. Comput. Electron. Agric. 207, 107746. doi: 10.1016/j.compag.2023.107746

[B15] Bossung C. Schlerf M. Machwitz M. (2022). Estimation of canopy nitrogen content in winter wheat from Sentinel-2 images for operational agricultural monitoring. Precis. Agric. 23, 2229–2252. doi: 10.1007/s11119-022-09918-y

[B16] Broadley M. Brown P. Cakmak I. Rengel Z. Zhao F. (2012). “Chapter 7 - Function of Nutrients: Micronutrients,” in Marschner’s Mineral Nutrition of Higher Plants, 3rd ed. Ed. Marschner P. (Academic Press, San Diego), 191–248. doi: 10.1016/B978-0-12-384905-2.00007-8

[B17] Burnett A. C. Anderson J. Davidson K. J. Ely K. S. Lamour J. Li Q.. (2021). A best-practice guide to predicting plant traits from leaf-level hyperspectral data using partial least squares regression. J. Exp. Bot. 72, 6175–6189. doi: 10.1093/jxb/erab295 34131723

[B18] Chan J. Y.-L. Leow S. M. H. Bea K. T. Cheng W. K. Phoong S. W. Hong Z.-W.. (2022). Mitigating the multicollinearity problem and its machine learning approach: A review. Mathematics 10, 1283. doi: 10.3390/math10081283

[B19] Chang C.-W. Laird D. A. Mausbach M. J. Hurburgh C. R. (2001). Near-infrared reflectance spectroscopy–principal components regression analyses of soil properties. Soil Sci. Soc. America J. 65, 480–490. doi: 10.2136/sssaj2001.652480x

[B20] Cherif E. Feilhauer H. Berger K. Dao P. D. Ewald M. Hank T. B.. (2023). From spectra to plant functional traits: Transferable multi-trait models from heterogeneous and sparse data. Remote Sens. Environ. 292, 113580. doi: 10.1016/j.rse.2023.113580

[B21] Chong I.-G. Jun C.-H. (2005). Performance of some variable selection methods when multicollinearity is present. Chemometrics Intelligent Lab. Syst. 78, 103–112. doi: 10.1016/j.chemolab.2004.12.011

[B22] Chungcharoen T. Donis-Gonzalez I. Phetpan K. Udompetaikul V. Sirisomboon P. Suwalak R. (2022). Machine learning-based prediction of nutritional status in oil palm leaves using proximal multispectral images. Comput. Electron. Agric. 198, 107019. doi: 10.1016/j.compag.2022.107019

[B23] Clay D. E. Kharel T. P. Reese C. Beck D. Carlson C. G. Clay S. A.. (2012). Winter wheat crop reflectance and nitrogen sufficiency index values are influenced by nitrogen and water stress. Agronomy Journal 104(6), 1612–1617. doi: 10.2134/agronj2012.0216

[B24] Curran P. J. (1989). Remote sensing of foliar chemistry. Remote Sens. Environ. 30, 271–278. doi: 10.1016/0034-4257(89)90069-2

[B25] Danner M. Locherer M. Hank T. Richter K. (2015). Spectral Sampling with the ASD FIELDSPEC 4. EnMAP Field Guides Technical Report. (Potsdam, Germany: GFZ Data Services). doi: 10.2312/ENMAP.2015.008

[B26] Das B. Manohara K. K. Mahajan G. R. Sahoo R. N. (2020a). Spectroscopy based novel spectral indices, PCA- and PLSR-coupled machine learning models for salinity stress phenotyping of rice. Spectrochimica Acta Part A: Mol. Biomolecular Spectrosc. 229, 117983. doi: 10.1016/j.saa.2019.117983 31896051

[B27] Das B. Sahoo R. N. Pargal S. Krishna G. Verma R. Chinnusamy V.. (2020b). Comparative analysis of index and chemometric techniques-based assessment of leaf area index (LAI) in wheat through field spectroradiometer, Landsat-8, Sentinel-2 and Hyperion bands. Geocarto Int. 35, 1415–1432. doi: 10.1080/10106049.2019.1581271

[B28] de Jong S. (1993). SIMPLS: An alternative approach to partial least squares regression. Chemometrics Intelligent Lab. Syst. 18, 251–263. doi: 10.1016/0169-7439(93)85002-X

[B29] Doughty C. E. Asner G. P. Martin R. E. (2011). Predicting tropical plant physiology from leaf and canopy spectroscopy. Oecologia 165, 289–299. doi: 10.1007/s00442-010-1800-4 20963611

[B30] Ecarnot M. Compan F. Roumet P. (2013). Assessing leaf nitrogen content and leaf mass per unit area of wheat in the field throughout plant cycle with a portable spectrometer. Field Crops Res. 140, 44–50. doi: 10.1016/j.fcr.2012.10.013

[B31] Ennaji O. Vergütz L. El Allali A. (2023). Machine learning in nutrient management: A review. Artif. Intell. Agric. 9, 1–11. doi: 10.1016/j.aiia.2023.06.001

[B32] Erenstein O. Jaleta M. Mottaleb K. A. Sonder K. Donovan J. Braun H.-J. (2022). “Global Trends in Wheat Production, Consumption and Trade,” in Wheat Improvement: Food Security in a Changing Climate. Eds. Reynolds M. P. Braun H.-J. (Springer International Publishing, Cham), 47–66. doi: 10.1007/978-3-030-90673-3_4

[B33] Fageria N. K. Baligar V. C. Clark R. B. (2002). Micronutrients in crops. Ad. Agron. 77, 185–268. doi: 10.1016/S0065-2113(02)77015-6

[B34] Fan X. Zhou X. Chen H. Tang M. Xie X. (2021). Cross-talks between macro- and micronutrient uptake and signaling in plants. Front. Plant Sci. 12. doi: 10.3389/fpls.2021.663477 PMC8555580 34721446

[B35] Flynn K. C. Frazier A. E. Admas S. (2020). Nutrient Prediction for Tef (Eragrostis tef) Plant and Grain with Hyperspectral Data and Partial Least Squares Regression: Replicating Methods and Results across Environments. Remote Sens. 12, 2867. doi: 10.3390/rs12182867

[B36] Furlanetto R. H. Crusiol L. G. T. Gonçalves J. V. F. Nanni M. R. de Oliveira Junior A. de Oliveira F. A.. (2023). Machine learning as a tool to predict potassium concentration in soybean leaf using hyperspectral data. Precis. Agric. 24, 2264–2292. doi: 10.1007/s11119-023-10040-w

[B37] Fushiki T. (2011). Estimation of prediction error by using K-fold cross-validation. Stat. Comput. 21, 137–146. doi: 10.1007/s11222-009-9153-8

[B38] Gara T. W. Skidmore A. K. Darvishzadeh R. Wang T. (2019). Leaf to canopy upscaling approach affects the estimation of canopy traits. GISci. Remote Sens. 56, 554–575. doi: 10.1080/15481603.2018.1540170

[B39] Geladi P. Kowalski B. R. (1986). Partial least-squares regression: a tutorial. Analytica Chimica Acta 185, 1–17. doi: 10.1016/0003-2670(86)80028-9

[B40] Grieco M. Schmidt M. Warnemünde S. Backhaus A. Klück H.-C. Garibay A.. (2022). Dynamics and genetic regulation of leaf nutrient concentration in barley based on hyperspectral imaging and machine learning. Plant Sci. 315, 111123. doi: 10.1016/j.plantsci.2021.111123 35067296

[B41] Gurudatta B. N. V. Sachan S. (2020). Effect of macro and micronutrients on growth of wheat crop- A review. Int. J. Innovative Res. Technol. 7, 221–227.

[B42] Hansen P. M. Schjoerring J. K. (2003). Reflectance measurement of canopy biomass and nitrogen status in wheat crops using normalized difference vegetation indices and partial least squares regression. Remote Sens. Environ. 86, 542–553. doi: 10.1016/S0034-4257(03)00131-7

[B43] Hawkesford M. J. Cakmak I. Coskun D. De Kok L. J. Lambers H. Schjoerring J. K.. (2023). “Functions of macronutrients☆,” in Marschner’s Mineral Nutrition of Plants, 4th ed. Eds. Rengel Z. Cakmak I. White P. J. (Academic Press, San Diego), 201–281. doi: 10.1016/B978-0-12-819773-8.00019-8

[B44] Hernandez J. Lobos G. A. Matus I. Del Pozo A. Silva P. Galleguillos M. (2015). Using ridge regression models to estimate grain yield from field spectral data in bread wheat (Triticum aestivum L.) grown under three water regimes. Remote Sens. 7, 2109–2126. doi: 10.3390/rs70202109

[B45] Hubert M. Branden K. V. (2003). Robust methods for partial least squares regression. J. Chemometrics 17, 537–549. doi: 10.1002/cem.822

[B46] Hunt R. Wang C. Booth D. Cox S. Kumar L. Reeves M. (2015). Remote Sensing of Rangeland Biodiversity. (Boca Raton, USA: Taylor & Francis Group LLC.), 277–307.

[B47] Im J. Jensen J. R. (2008). Hyperspectral remote sensing of vegetation. Geogr. Compass 2, 1943–1961. doi: 10.1111/j.1749-8198.2008.00182.x

[B48] Jamali M. Soufizadeh S. Yeganeh B. Emam Y. (2023). Wheat leaf traits monitoring based on machine learning algorithms and high-resolution satellite imagery. Ecol. Inf. 74, 101967. doi: 10.1016/j.ecoinf.2022.101967

[B49] Jones J. B. Sr. Wolf B. Mills H. A. (1991). Microwave digestion using CEM microwave digestion system. Plant Analysis Handbook (Athens, GA: Micro-Macro Publishing).

[B50] Jordan M. I. Mitchell T. M. (2015). Machine learning: Trends, perspectives, and prospects. Science 349, 255–260. doi: 10.1126/science.aaa8415 26185243

[B51] Kalra Y. (1997). Handbook of Reference Methods for Plant Analysis (Boca Raton, FL, USA: CRC Press).

[B52] Khalid A. Hameed A. Tahir M. F. (2023). Wheat quality: A review on chemical composition, nutritional attributes, grain anatomy, types, classification, and function of seed storage proteins in bread making quality. Front. Nutr. 10. doi: 10.3389/fnut.2023.1053196 PMC9998918 36908903

[B53] Khan F. Siddique A. B. Shabala S. Zhou M. Zhao C. (2023). Phosphorus plays key roles in regulating plants’ Physiological responses to abiotic stresses. Plants (Basel) 12, 2861. doi: 10.3390/plants12152861 37571014 PMC10421280

[B54] Kokaly R. F. Asner G. P. Ollinger S. V. Martin M. E. Wessman C. A. (2009). Characterizing canopy biochemistry from imaging spectroscopy and its application to ecosystem studies. Remote Sens. Environment Imaging Spectrosc. Special Issue 113, S78–S91. doi: 10.1016/j.rse.2008.10.018

[B55] Kothari S. Beauchamp-Rioux R. Blanchard F. Crofts A. L. Girard A. Guilbeault-Mayers X.. (2023). Predicting leaf traits across functional groups using reflectance spectroscopy. New Phytol. 238, 549–566. doi: 10.1111/nph.18713 36746189

[B56] Kovar M. Brestic M. Sytar O. Barek V. Hauptvogel P. Zivcak M. (2019). Evaluation of hyperspectral reflectance parameters to assess the leaf water content in soybean. Water 11, 443. doi: 10.3390/w11030443

[B57] Li L. Lin D. Wang J. Yang L. Wang Y. (2020). Multivariate analysis models based on full spectra range and effective wavelengths using different transformation techniques for rapid estimation of leaf nitrogen concentration in winter wheat. Front. Plant Sci. 11. doi: 10.3389/fpls.2020.00755 PMC7333249 32676083

[B58] Lyu H. Grafton M. Ramilan T. Irwin M. Sandoval E. (2023). Assessing the leaf blade nutrient status of pinot noir using hyperspectral reflectance and machine learning models. Remote Sens. 15, 1497. doi: 10.3390/rs15061497

[B59] Mahajan G. R. Das B. Kumar P. Murgaokar D. Patel K. Desai A.. (2024). Spectroscopy-based chemometrics combined machine learning modeling predicts cashew foliar macro- and micronutrients. Spectrochimica Acta Part A: Mol. Biomolecular Spectrosc. 320, 124639. doi: 10.1016/j.saa.2024.124639 38878723

[B60] Mahajan G. R. Das B. Murgaokar D. Herrmann I. Berger K. Sahoo R. N.. (2021). Monitoring the foliar nutrients status of mango using spectroscopy-based spectral indices and PLSR-combined machine learning models. Remote Sens. 13. doi: 10.3390/rs13040641

[B61] Mahajan G. R. Sahoo R. N. Pandey R. N. Gupta V. K. Kumar D. (2014). Using hyperspectral remote sensing techniques to monitor nitrogen, phosphorus, sulphur and potassium in wheat (Triticum aestivum L.). Precis. Agric. 15, 499–522. doi: 10.1007/s11119-014-9348-7

[B62] Malvern Panalytical (2023). Malvern panalytical. Available online at: https://www.malvernpanalytical.com/en/products/product-range/asd-range/fieldspec-range/fieldspec-4-standard-res-spectroradiometer (Accessed 4.20.24)).

[B63] Meacham K. Fu P. Wu J. Serbin S. Montes C. Ainsworth E.. (2020). Plot level rapid screening for photosynthetic parameters using proximal hyperspectral imaging. J. Exp. Bot. 71, 2312–2328. doi: 10.1093/jxb/eraa068 32092145 PMC7134947

[B64] Mehmood T. Liland K. H. Snipen L. Sæbø S. (2012). A review of variable selection methods in Partial Least Squares Regression. Chemometrics Intelligent Lab. Syst. 118, 62–69. doi: 10.1016/j.chemolab.2012.07.010

[B65] Mehmood T. Sæbø S. Liland K. H. (2020). Comparison of variable selection methods in partial least squares regression. J. Chemometrics 34. doi: 10.1002/cem.3226

[B66] Menesatti P. Antonucci F. Pallottino F. Roccuzzo G. Allegra M. Stagno F.. (2010). Estimation of plant nutritional status by Vis–NIR spectrophotometric analysis on orange leaves [*Citrus sinensis* (L) Osbeck *cv* Tarocco. Biosyst. Eng. 105, 448–454. doi: 10.1016/j.biosystemseng.2010.01.003

[B67] Molnar C. (2020). Interpretable Machine Learning (Morrisville, NC: Lulu.com).

[B68] Montesinos-López O. A. Montesinos-López A. Crossa J. de los Campos G. Alvarado G. Suchismita M.. (2017). Predicting grain yield using canopy hyperspectral reflectance in wheat breeding data. Plant Methods 13, 4. doi: 10.1186/s13007-016-0154-2 28053649 PMC5209864

[B69] Morshedloo M. R. Salami S. A. Nazeri V. Craker L. E. (2016). Prolonged water stress on growth and constituency of Iranian of oregano (Origanum vulgare L.). Journal of Medicinally Active Plants 5, 7–19. doi: 10.7275/R5XS5SKW

[B70] Nakaji T. Oguma H. Nakamura M. KaChina P. Asanok L. Marod D.. (2019). Estimation of six leaf traits of East Asian forest tree species by leaf spectroscopy and partial least square regression. Remote Sens. Environ. 233, 111381. doi: 10.1016/j.rse.2019.111381

[B71] Nduku L. Munghemezulu C. Mashaba-Munghemezulu Z. Kalumba A. M. Chirima G. J. Masiza W.. (2023). Global research trends for unmanned aerial vehicle remote sensing application in wheat crop monitoring. Geomatics 3, 115–136. doi: 10.3390/geomatics3010006

[B72] Ng C. Q. J. Toh Y. Y. Lam C. Y. L. Chang C. W. Liew S. C. (2007). Effects of leaf water content on reflectance. 28th Asian Conference on Remote Sensing (ACRS) 2007, Vol. 1.

[B73] Oliveira L. F. R. Santana R. C. (2020). Estimation of leaf nutrient concentration from hyperspectral reflectance in Eucalyptus using partial least squares regression. Sci. Agric. (Piracicaba Braz.) 77, e20180409. doi: 10.1590/1678-992X-2018-0409

[B74] Oliveira L. F. R. Santana R. C. de M. L. R. (2019). Non destructive estimation of leaf nutrient concentrations in eucalyptus plantations. CERNE 25, 184–194. doi: 10.1590/01047760201925022631

[B75] Osco L. P. Junior J. M. Ramos A. P. M. Furuya D. E. G. Santana D. C. Teodoro L. P. R.. (2020a). Leaf nitrogen concentration and plant height prediction for maize using UAV-based multispectral imagery and machine learning techniques. Remote Sens. 12, 3237. doi: 10.3390/rs12193237

[B76] Osco L. P. Ramos A. P. M. Faita Pinheiro M. M. Moriya É.A.S. Imai N. N. Estrabis N.. (2020b). A machine learning framework to predict nutrient content in valencia-orange leaf hyperspectral measurements. Remote Sens. 12, 906. doi: 10.3390/rs12060906

[B77] Pandey M. Shrestha J. Subedi S. Shah K. K. (2020). Role of nutrients in wheat: A review. Trop.agr.bio. 1, 18–23. doi: 10.26480/trab.01.2020.18.23

[B78] Panigrahi N. Das B. S. (2021). Evaluation of regression algorithms for estimating leaf area index and canopy water content from water stressed rice canopy reflectance. Inf. Process. Agric. 8, 284–298. doi: 10.1016/j.inpa.2020.06.002

[B79] Pimstein A. Karnieli A. Bansal S. K. Bonfil D. J. (2011). Exploring remotely sensed technologies for monitoring wheat potassium and phosphorus using field spectroscopy. Field Crops Res. 121, 125–135. doi: 10.1016/j.fcr.2010.12.001

[B80] Pimstein A. Karnieli A. Bonfil D. (2007). Wheat and maize monitoring based on ground spectral measurements and multivariate data analysis. JARS 1, 013530. doi: 10.1117/1.2784799

[B81] Prananto J. A. Minasny B. Weaver T. (2020). Near infrared (NIR) spectroscopy as a rapid and cost-effective method for nutrient analysis of plant leaf tissues. Adv. Agron. 164, 1–49. doi: 10.3389/fpls.2021.809828

[B82] Prasad B. Babar M. A. Carver B. F. Raun W. R. Klatt A. R. (2009). Association of biomass production and canopy spectral reflectance indices in winter wheat. Can. J. Plant Sci. 89, 485–496. doi: 10.4141/CJPS08137

[B83] Prasad B. Carver B. F. Stone M. L. Babar M. A. Raun W. R. Klatt A. R. (2007). Potential use of spectral reflectance indices as a selection tool for grain yield in winter wheat under great plains conditions. Crop Sci. 47, 1426–1440. doi: 10.2135/cropsci2006.07.0492

[B84] Pullanagari R. R. Kereszturi G. Yule I. J. (2016). Mapping of macro and micro nutrients of mixed pastures using airborne AisaFENIX hyperspectral imagery. ISPRS J. Photogrammetry Remote Sens. 117, 1–10. doi: 10.1016/j.isprsjprs.2016.03.010

[B85] Raya-Sereno M. D. Ortiz-Monasterio J. I. Alonso-Ayuso M. Rodrigues F. A. Rodríguez A. A. González-Perez L.. (2021). High-resolution airborne hyperspectral imagery for assessing yield, biomass, grain N concentration, and N output in spring wheat. Remote Sens. 13, 1373. doi: 10.3390/rs13071373

[B86] Robles-Zazueta C. A. Pinto F. Molero G. Foulkes M. J. Reynolds M. P. Murchie E. H. (2022). Prediction of photosynthetic, biophysical, and biochemical traits in wheat canopies to reduce the phenotyping bottleneck. Front. Plant Sci. 13. doi: 10.3389/fpls.2022.828451 PMC9036448 35481146

[B87] Sawatsky M. L. Clyde M. Meek F. (2015). Partial least squares regression in the social sciences. TQMP 11, 52–62. doi: 10.20982/tqmp.11.2.p052

[B88] Serbin S. P. Singh A. McNeil B. E. Kingdon C. C. Townsend P. A. (2014). Spectroscopic determination of leaf morphological and biochemical traits for northern temperate and boreal tree species. Ecol. Appl. 24, 1651–1669. doi: 10.1890/13-2110.1 29210229

[B89] Serbin S. P. Wu J. Ely K. S. Kruger E. L. Townsend P. A. Meng R.. (2019). From the Arctic to the tropics: multibiome prediction of leaf mass per area using leaf reflectance. New Phytol. 224, 1557–1568. doi: 10.1111/nph.16123 31418863

[B90] Shakoor N. Lee S. Mockler T. C. (2017). High throughput phenotyping to accelerate crop breeding and monitoring of diseases in the field. Curr. Opin. Plant Biol. 38, 184–192. doi: 10.1016/j.pbi.2017.05.006 28738313

[B91] Singh A. Serbin S. P. McNeil B. E. Kingdon C. C. Townsend P. A. (2015). Imaging spectroscopy algorithms for mapping canopy foliar chemical and morphological traits and their uncertainties. Ecol. Appl. 25, 2180–2197. doi: 10.1890/14-2098.1 26910948

[B92] Singh H. Roy A. Setia R. K. Pateriya B. (2022). Estimation of nitrogen content in wheat from proximal hyperspectral data using machine learning and explainable artificial intelligence (XAI) approach. Model. Earth Syst. Environ. 8, 2505–2511. doi: 10.1007/s40808-021-01243-z

[B93] Singh L. Mutanga O. Mafongoya P. Peerbhay K. Crous J. (2022). Hyperspectral remote sensing for foliar nutrient detection in forestry: A near-infrared perspective. Remote Sens. Applications: Soc. Environ. 25, 100676. doi: 10.1016/j.rsase.2021.100676

[B94] Stein B. R. Thomas V. A. Lorentz L. J. Strahm B. D. (2014). Predicting macronutrient concentrations from loblolly pine leaf reflectance across local and regional scales. GISci. Remote Sens. 51, 269–287. doi: 10.1080/15481603.2014.912875

[B95] Sticksel E. Schächtl J. Huber G. Liebler J. Maidl F.-X. (2004). Diurnal variation in hyperspectral vegetation indices related to winter wheat biomass formation. Precis. Agric. 5, 509–520. doi: 10.1007/s11119-004-5322-0

[B96] Sze V. Chen Y.-H. Yang T.-J. Emer J. S. (2017). Efficient processing of deep neural networks: A tutorial and survey. Proc. IEEE 105, 2295–2329. doi: 10.1109/JPROC.2017.2761740

[B97] Thompson D. R. Bohn N. Brodrick P. G. Carmon N. Eastwood M. L. Eckert R.. (2022). Atmospheric lengthscales for global VSWIR imaging spectroscopy. J. Geophys. Res. Biogeosci. 127, e2021JG006711. doi: 10.1029/2021JG006711 PMC9285454 35859986

[B98] Thorp K. R. Wang G. Bronson K. F. Badaruddin M. Mon J. (2017). Hyperspectral data mining to identify relevant canopy spectral features for estimating durum wheat growth, nitrogen status, and grain yield. Comput. Electron. Agric. 136, 1–12. doi: 10.1016/j.compag.2017.02.024

[B99] Tobias R. D. (2000). An Introduction to Partial Least Squares Regression. (Cary, NC, USA: SAS Institute Inc.).

[B100] Tran T. Szymańska E. Gerretzen J. Buydens L. Afanador N. L. Blanchet L. (2017). Weight randomization test for the selection of the number of components in PLS models. J. Chemometrics 31, e2887. doi: 10.1002/cem.2887

[B101] Ustin S. L. Jacquemoud S. (2020). “How the Optical Properties of Leaves Modify the Absorption and Scattering of Energy and Enhance Leaf Functionality,” in Remote Sensing of Plant Biodiversity. Eds. Cavender-Bares J. Gamon J. A. Townsend P. A. (Springer International Publishing, Cham), 349–384. doi: 10.1007/978-3-030-33157-3_14

[B102] Van der Voet H. (1994). Comparing the predictive accuracy of models using a simple randomization test. Chemometrics Intelligent Lab. Syst. 25, 313–323. doi: 10.1016/0169-7439(94)85050-X

[B103] Wang J. Wang T. Skidmore A. K. Shi T. Wu G. (2015). Evaluating different methods for grass nutrient estimation from canopy hyperspectral reflectance. Remote Sens. 7, 5901–5917. doi: 10.3390/rs70505901

[B104] Wang M. Zheng Q. Shen Q. Guo S. (2013). The critical role of potassium in plant stress response. Int. J. Mol. Sci. 14, 7370–7390. doi: 10.3390/ijms14047370 23549270 PMC3645691

[B105] Wang S. Guan K. Wang Z. Ainsworth E. A. Zheng T. Townsend P. A.. (2021). Unique contributions of chlorophyll and nitrogen to predict crop photosynthetic capacity from leaf spectroscopy. J. Exp. Bot. 72, 341–354. doi: 10.1093/jxb/eraa432 32937655

[B106] Wang Z. Chlus A. Geygan R. Ye Z. Zheng T. Singh A.. (2020). Foliar functional traits from imaging spectroscopy across biomes in eastern North America. New Phytol. 228, 494–511. doi: 10.1111/nph.16711 32463927

[B107] Weissert C. Kehr J. (2017). Macronutrient sensing and signaling in plants. In Plant macronutrient use efficiency (Cambridge, MA, USA: Academic Press), 45–64.

[B108] Welch R. M. Shuman L. (1995). Micronutrient nutrition of plants. Crit. Rev. Plant Sci. 14, 49–82. doi: 10.1080/07352689509701922

[B109] White P. J. Broadley M. R. (2003). Calcium in plants. Ann. Bot. 92, 487–511. doi: 10.1093/aob/mcg164 12933363 PMC4243668

[B110] Wold S. Sjöström M. Eriksson L. (2001). PLS-regression: a basic tool of chemometrics. Chemometrics Intelligent Lab. Syst. 58, 109–130. doi: 10.1016/S0169-7439(01)00155-1

[B111] Xie Y. Wang C. Yang W. Feng M. Qiao X. Song J. (2020). Canopy hyperspectral characteristics and yield estimation of winter wheat (Triticum aestivum) under low temperature injury. Sci. Rep. 10, 244. doi: 10.1038/s41598-019-57100-8 31937859 PMC6959340

[B112] Yang T. Lu J. Liao F. Qi H. Yao X. Cheng T.. (2021). Retrieving potassium levels in wheat blades using normalised spectra. Int. J. Appl. Earth Observation Geoinformation 102, 102412. doi: 10.1016/j.jag.2021.102412

[B113] Yoder B. J. Pettigrew-Crosby R. E. (1995). Predicting nitrogen and chlorophyll content and concentrations from reflectance spectra (400–2500 nm) at leaf and canopy scales. Remote Sens. Environ. 53, 199–211. doi: 10.1016/0034-4257(95)00135-N

[B114] Zahir S. A. D. M. Jamlos M. F. Omar A. F. Jamlos M. A. Mamat R. Muncan J.. (2024). Review – Plant nutritional status analysis employing the visible and near-infrared spectroscopy spectral sensor. Spectrochimica Acta Part A: Mol. Biomolecular Spectrosc. 304, 123273. doi: 10.1016/j.saa.2023.123273 37666099

[B115] Zaji A. Liu Z. Xiao G. Sangha J. S. Ruan Y. (2022). A survey on deep learning applications in wheat phenotyping. Appl. Soft Computing 131, 109761. doi: 10.1016/j.asoc.2022.109761

[B116] Zhai Y. Cui L. Zhou X. Gao Y. Fei T. Gao W. (2012). Estimation of nitrogen, phosphorus, and potassium contents in the leaves of different plants using laboratory-based visible and near-infrared reflectance spectroscopy: comparison of partial least-square regression and support vector machine regression methods. Int. J. Remote Sens. 34, 2502–2518. doi: 10.1080/01431161.2012.746484

[B117] Zhang P.-P. Zhou X.-X. Wang Z.-X. Mao W. Li W.-X. Yun F.. (2020). Using HJ-CCD image and PLS algorithm to estimate the yield of field-grown winter wheat. Sci. Rep. 10, 5173. doi: 10.1038/s41598-020-62125-5 32198471 PMC7083868

[B118] Zhang S. Duan J. Qi X. Gao Y. He L. Liu L.. (2024). Combining spectrum, thermal, and texture features using machine learning algorithms for wheat nitrogen nutrient index estimation and model transferability analysis. Comput. Electron. Agric. 222, 109022. doi: 10.1016/j.compag.2024.109022

[B119] Zhao D. Reddy K. R. Kakani V. G. Reddy V. R. (2005). Nitrogen deficiency effects on plant growth, leaf photosynthesis, and hyperspectral reflectance properties of sorghum. Eur. J. Agron. 22, 391–403. doi: 10.1016/j.eja.2004.06.005

[B120] Žibrat U. Širca S. Susič N. Knapič M. Gerič Stare B. Urek G. (2020). Noninvasive detection of plant parasitic nematodes using hyperspectral and other remote sensing systems. In Hyperspectral Remote Sensing (Elsevier), pp. 357–375.

