# Supplementary material for: Utilizing VSWIR spectroscopy for macronutrient and micronutrient profiling in winter wheat
Source: Front Plant Sci. 2024 Oct 31;15:1426077. doi: 10.3389/fpls.2024.1426077 (PMC11560459; doi:10.3389/fpls.2024.1426077)
Supplement: Supplementary file 1 [file DataSheet1.docx]

**Utilizing VSWIR spectroscopy for macronutrient and micronutrient profiling in winter wheat**

Anmol Kaur Gill^1^, Srishti Gaur^1^, Clay Sneller^2^, Darren T. Drewry^1,3,4*^

^1^Department of Food, Agricultural, and Biological Engineering, Ohio State University, Columbus, OH, USA

^2^Department of Horticulture and Crop Science, Ohio State University, Wooster, OH, USA

^3^Department of Horticulture and Crop Science, Ohio State University, Columbus, OH, USA

^4^Translational Data Analytics Institute, Ohio State University, Columbus, OH, USA

*** Correspondence:**Darren T. Drewry
drewry.19@osu.edu

**Supplementary Material**

**Figure S1. (A)** Histograms showing the distributions of the measured leaf nutrient concentrations of wheat crop. The blue bars represent the frequency of samples falling within specific concentration ranges, offering insights into central tendencies and variabilities. Superimposed on each histogram is a red solid line, depicting a fitted density curve. **(B)** This plot shows the mean leaf reflectance spectra across six sampling dates during the study period.

**
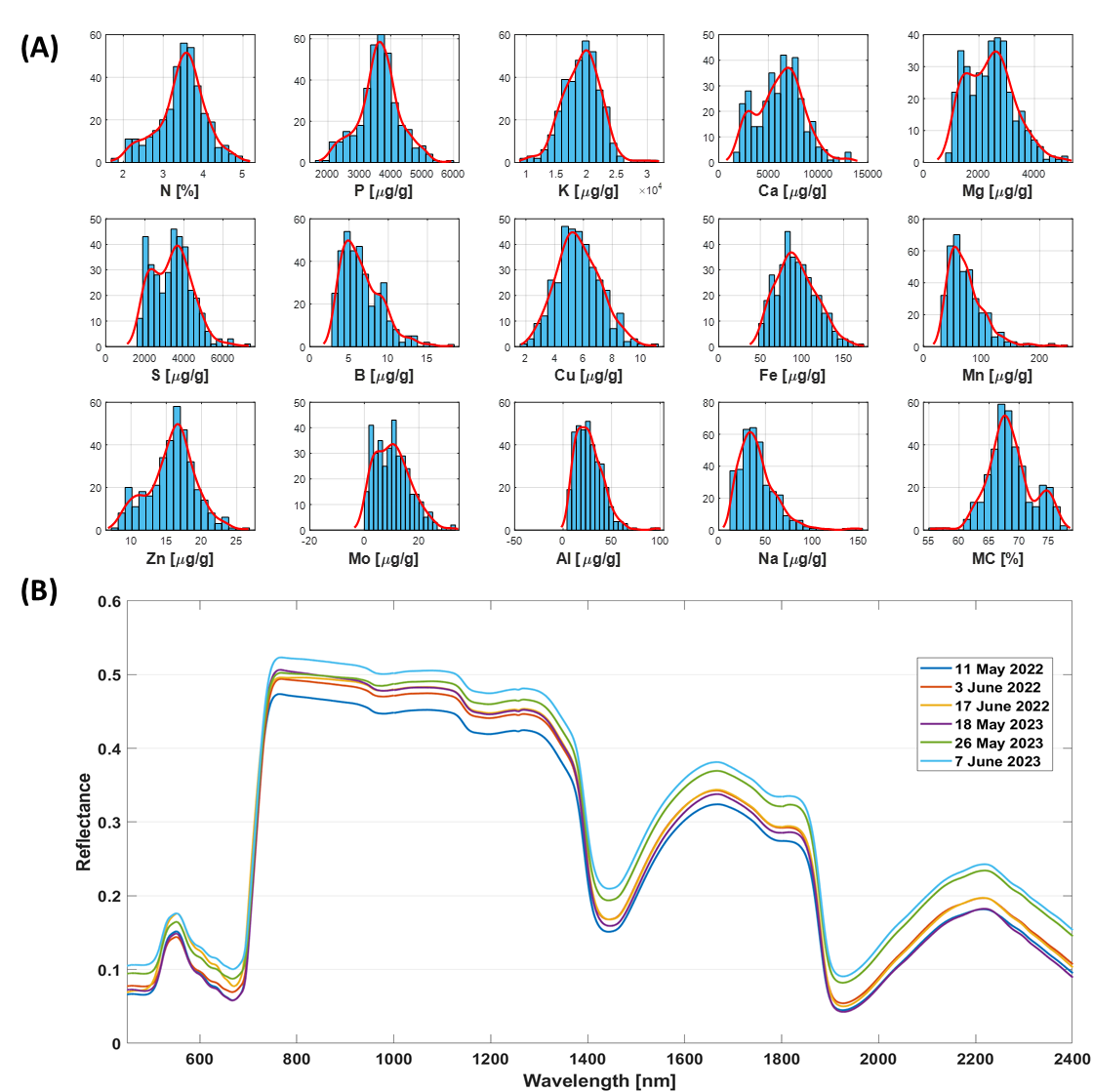
**

**Figure S2.** The figure comprises three subplots that collectively showcase the model selection and coefficient analysis process for the prediction of foliar nitrogen content. In **subplot** **(A)**, the blue line represents the calibration R^2^, illustrating the model's goodness of fit across a range of component numbers (1 to 30). Simultaneously, the red line signifies the validation R^2^. **Subplot** **(B)** portrays the Prediction Error Sum of Squares (PRESS) values, again in blue for calibration and red for validation, as they vary across different numbers of retained components. Notably, solid lines in black (Adjusted PRESS), green (Minimum PRESS), and magenta (Van der Voet's t-test) denote the optimal component selection methods. **Subplot (C)** presents standardized PLSR coefficients for the three previously mentioned approaches. These coefficients span the 450-2400 nm wavelength range. Please note that analogous figures exist (S2 through S16) for all the nutrients, with the same framework and legends, allowing for a comprehensive assessment of model selection and coefficient analysis across all nutrients in our study.

**Figure S3.** Same as Figure S2 for phosphorus.

**Figure S4.** Same as Figure S2 for potassium.

**Figure S5.** Same as Figure S2 for calcium.

**Figure S6.** Same as Figure S2 for magnesium.

**Figure S7.** Same as Figure S2 for sulfur.

**Figure S8.** Same as Figure S2 for boron.

**Figure S9.** Same as Figure S2 for copper.

**Figure S10.** Same as Figure S2 for iron.

**Figure S11.** Same as Figure S2 for manganese.

**Figure S12.** Same as Figure S2 for zinc.

**Figure S13.** Same as Figure S2 for molybdenum.

**Figure S14.** Same as Figure S2 for aluminum.

**Figure S15.** Same as Figure S2 for sodium.

**Figure S16.** Same as Figure S2 for water content.
